# Molecular basis of HHQ biosynthesis: molecular dynamics simulations, enzyme kinetic and surface plasmon resonance studies

**DOI:** 10.1186/2046-1682-6-10

**Published:** 2013-08-01

**Authors:** Anke Steinbach, Christine K Maurer, Elisabeth Weidel, Claudia Henn, Christian Brengel, Rolf W Hartmann, Matthias Negri

**Affiliations:** 1 Helmholtz-Institute for Pharmaceutical Research Saarland, Campus C2.3, 66123, Saarbrücken, Germany; 2 Pharmaceutical and Medicinal Chemistry, Saarland University, Campus C2.3, 66123, Saarbrücken, Germany; 3 ElexoPharm GmbH, Im Stadtwald A1.2, 66123, Saarbrücken, Germany; 4 Current address: MIP Pharma GmbH, Kirkelerstr. 41, 66440,Blieskastel-Niederwürzbach, Germany

## Abstract

**Background:**

PQS (*Pseudomonas*Quinolone Signal) and its precursor HHQ are signal molecules of the *P*. *aeruginosa quorum sensing* system. They explicate their role in mammalian pathogenicity by binding to the receptor PqsR that induces virulence factor production and biofilm formation. The enzyme PqsD catalyses the biosynthesis of HHQ.

**Results:**

Enzyme kinetic analysis and surface plasmon resonance (SPR) biosensor experiments were used to determine mechanism and substrate order of the biosynthesis. Comparative analysis led to the identification of domains involved in functionality of PqsD. A kinetic cycle was set up and molecular dynamics (MD) simulations were used to study the molecular bases of the kinetics of PqsD. Trajectory analysis, pocket volume measurements, binding energy estimations and decompositions ensured insights into the binding mode of the substrates anthraniloyl-CoA and β-ketodecanoic acid.

**Conclusions:**

Enzyme kinetics and SPR experiments hint at a ping-pong mechanism for PqsD with ACoA as first substrate. Trajectory analysis of different PqsD complexes evidenced ligand-dependent induced-fit motions affecting the modified ACoA funnel access to the exposure of a secondary channel. A tunnel-network is formed in which Ser317 plays an important role by binding to both substrates. Mutagenesis experiments resulting in the inactive S317F mutant confirmed the importance of this residue. Two binding modes for β-ketodecanoic acid were identified with distinct catalytic mechanism preferences.

## Background

*Quorum sensing* (QS) is a chemical cell-to-cell communication system in bacteria ruled by small extracellular signal molecules. It coordinates the social life of bacteria by regulating many group-related behaviours, such as biofilm formation and virulence factor production [1-5]. Anti-QS has been recognized as an attractive strategy in the fight against bacteria [6] based on anti-virulence and anti-biofilm action and not on bacterial killing.

The opportunistic Gram-negative pathogen *P*. *aeruginosa* is a good model to study the complexity of QS systems [1,4]. At least three distinct QS pathways are known which regulate in a hierarchical manner the QS-dependent target gene expression. The first two QS systems, *las*[7] and *rhl*[8], utilize *N*-acyl homoserine lactones (C_4_- and C_12_-AHL) and the receptors LasR and RhlR [9]. The third QS-system is 2-alkyl-4-hydroxyquinoline (HAQ)-dependent and specific for *P*. *aeruginosa* and some *Burkholderia* strains [10-12]. PQS (*Pseudomonas*Quinolone Signal) and to a lesser extent its precursor HHQ (2-heptyl-4-hydroxyquinoline) activate PqsR [13-15].

A key enzyme of the PQS biosynthesis pathway is PqsD (PQB biosynthetic 3-oxoacyl-[acyl-carrier-protein] [ACP] synthase III; EC 2.3.1.180), which catalyses the formation of HHQ by “head-to-head” decarboxylative condensation of anthranilate (as anthraniloyl-CoA; ACoA) and β-ketodecanoic acid (βK) [16,17].

Several groups have proven that *P*. *aeruginosa pqsD* knock-out mutant as well as PQS-deficient *P*. *aeruginosa* strains have an attenuated pathogenicity in nematode and mouse models evidencing the significance of PQS signalling in mammalian pathogenesis [18]. Increased PQS levels have been detected in lungs of cystic fibrosis patients supportive for an active role of QS in chronic lung infections [19-21]. These findings and in particular the recent identification of the first class of PqsD inhibitors that reduce biofilm and virulence factor formation in *P*. *aeruginosa* validates PqsD as a target for the development of anti-infectives [22].

PqsD is a homodimeric bi-substrate enzyme with high structural similarity to FabH and other β-ketoacyl-[ACP] synthases III (KAS III). They share a common thiolase fold (αβαβα), a long tunnel to the active site, and the same catalytic residues [23-25]. Three PDB structures of PqsD exist [26]: as apoform (3H76), as Cys112-ligated anthranilate (CSJ) complex with ACoA molecules in the primary funnel (3H77) and as Cys112Ala mutant in complex with anthranilic acid (3H78) [23]. In all three structures the catalytic centre is accessible by two channels in L-shape: the primary CoA/ACP-funnel, and the shorter secondary channel (Additional file 1: Figure. SI1). However, the molecular details of ACoA access and, in particular, the binding mode and the subsequent incorporation of βK are unknown.

Knowledge of the kinetics and of the conformational flexibility of an enzyme can significantly contribute to a successful rational drug design [27-29]. Herein we study the molecular basis of PqsD and the HHQ biosynthesis combining experimental and *in silico* methods. Enzyme kinetic analysis and surface plasmon resonance (SPR) biosensor experiments were used to determine the mechanism and the substrate order of the biosynthesis; comparative analysis of PqsD to homologous KAS-III enzymes was useful to identify domains specific for PqsD functionality. Molecular dynamics (MD) simulations were carried out to explore the binding modes of ACoA and βK as well as the conformational flexibility of PqsD.

## Results and discussion

Knowledge of enzyme kinetics for multi-substrate reactions is helpful to set up and interpret MD simulations. We performed biochemical and biophysical studies to determine the underlying kinetic mechanism of PqsD.

### Biochemical and biophysical characterization hint at ping-pong kinetic mechanism of PqsD

**Figure 1:**
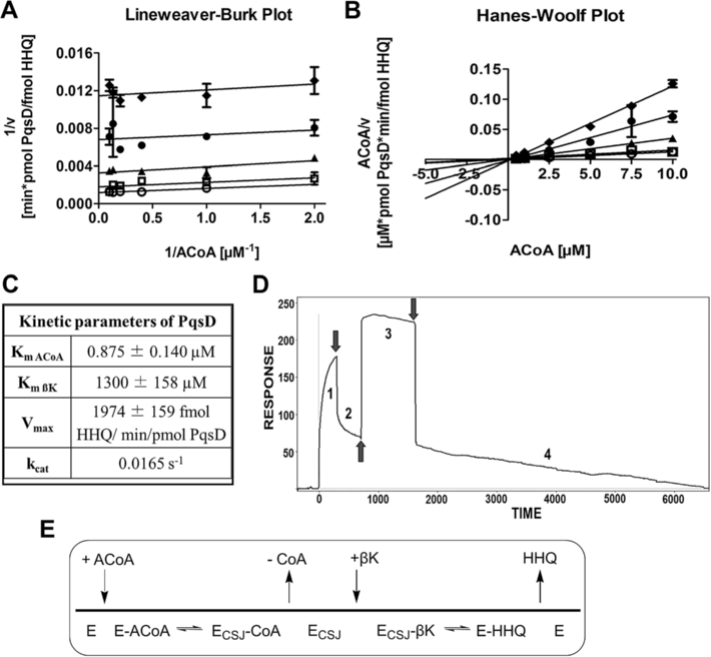
**Ping**-**pong kinetic mechanism of PqsD in HHQ biosynthesis. A)** Lineweaver-Burk Plot and **B)** Hanes-Woolf Plot representing the activity of PqsD as a function of ACoA concentration in the presence of 60 (diamonds), 120 (black circles), 240 (triangles), 480 (open squares) and 1000 μM (open circles) ßK. All data are mean values of four replicates. **C)** Kinetic parameters of PqsD. **D)** “Real-time” PqsD kinetics by SPR experiments: 1. ACoA injection; 2. buffer run - response units remain high due to anthranilate-Cys_112_; 3. βK injection; 4. Buffer run – response line goes to zero due to HHQ formation and restored apo PqsD. **E)** Schematic view of the putative ping-pong kinetic mechanism of the HHQ biosynthesis (E - PqsD, E_CSJ_ - PqsD with Cys112-ligated anthranilate).

Enzyme kinetic studies were performed using a 96-well format-based *in vitro* assay with the purified enzyme PqsD to determine the kinetic parameters for each substrate. Optimum enzymatic reaction conditions were determined in advance. Plotting product formation *versus* time revealed that a reaction time of 4 min in combination with an enzyme concentration of 0.25 μM was suitable and that values in linear range within the progress curve could be obtained (data not shown). The initial velocity (*v*) was calculated by dividing the product concentration by the reaction time. Plotting the data with GraphPad Prism 5 software resulted in an array of parallel lines in the Lineweaver-Burk-Plot and a common Y-axis-intercept in the Hanes-Woolf-Plot (Figure 1A and 1B). This suggests a ping pong kinetic mechanism for PqsD as described also for other Claisen condensing enzymes [30]. The results were in agreement with reported mass spectrometric [31], structural [23], and surface plasmon resonance (SPR) based [32] studies revealing the formation of an anthraniloyl-PqsD intermediate with concomitant release of the first product CoA before binding of the second substrate β-ketodecanoic acid (βK).

The *K*_M_ data (ACoA 0.875 ±0.140 μM, βK 1300 ±158 μM) correlate well with the *K*_D_ values determined with SPR by our group (ACoA 1.08 μM, βK 2.95 mM) [32]. Also, the kinetic parameters, derived mutually varying both substrates (Figure 1C) and fitting the data with the ping-pong Equation (1) are within the range of the apparent values determined by Pistorius et al. (*K*_M app,ßK_ = 598.5 ± 106 μM; V_max_ = 495.8 ± 37.5 fmol HHQ/min/pmol PqsD; *k*_cat (PqsD as monomer)_ = 0.01 s^-1^).$v=\mathrm{Vmax}\frac{\left[\mathrm{ACoA}\right]}{\mathrm{Km}\;\mathrm{ACoA}+\left[\mathrm{ACoA}\right]\;\left(1+\left(\frac{\mathrm{Km}\;\;\mathrm{\beta K}}{\left[\mathrm{\beta K}\right]}\right)\right)}$ (1) [33] SPR biosensor assays were performed to assess the influence of substrate addition order on the HHQ product formation. Firstly, as recently reported [32], PqsD was immobilized to the SPR chip and ACoA injected; the increase in the response-line preserved also after washing was an indicator for the covalent linkage of anthraniloyl to Cys112 (Figure 1D and Additional file 1: SI1). The subsequent addition of βK displaced the anthraniloyl from Cys112 with HHQ formation as confirmed by mass spectrometry (see supporting information Text SI2). Strikingly, repeating the experiments with inverted substrate order (βK first, then ACoA) resulted in less than half of HHQ formation (Additional file 1: Figure SI2A) supportive for the preferential substrate order deducible from the kinetic analysis of the HHQ biosynthesis. However, the latter finding cannot be excluded to be at least in part due to substrate inhibition. The different plots of the kinetic data and the “inverted” SPR experiments sustain the idea that PqsD follows a ping-pong kinetic mechanism with ACoA as the first substrate (Figure 1E). Based on these results a putative kinetic cycle for HHQ biosynthesis was set-up and different PqsD-ligand complexes chosen to simulate the single steps (Additional file 1: Figure. SI2B).

### Comparative analysis

**Figure 2:**
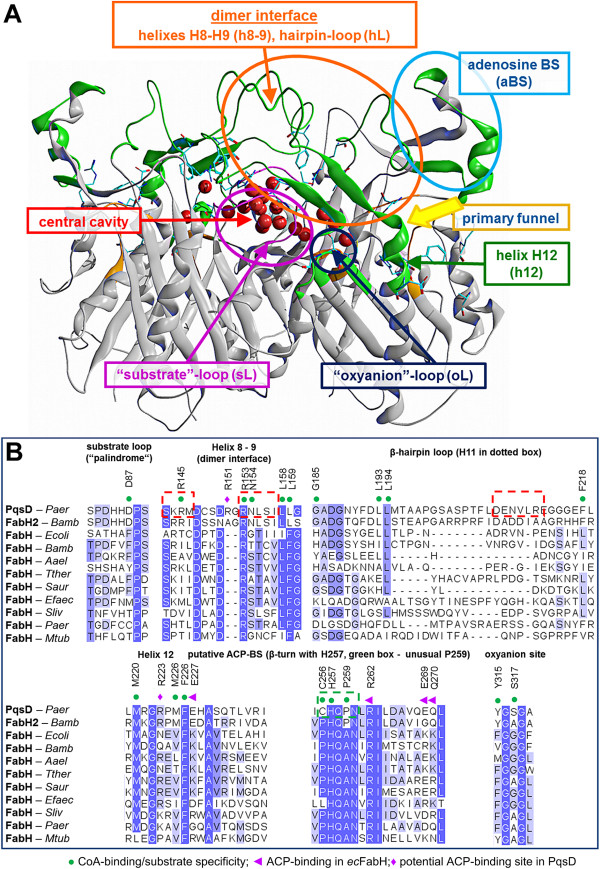
**Functionally important domains of PqsD. A)** 3D structure of the PqsD dimer with functionally significant domains and the central cavity. PqsD is rendered as cartoon with flexible regions coloured in green. Water molecules present in the central cavity are shown as red spheres. **B)** Multiple sequence alignment of PqsD and homologous KAS-III enzymes. Abbreviations: Paer – *Pseudomonas aeruginosa*, Bamb – *Burkholderia ambifaria*, Ecoli – *Escherichia coli*, Aael – *Aquifex aeolicus*, Tther – *Thermus thermophilus*, Saur – *Staphylococcus aureus*, Efaec – *Enterococcus faecalis*, Sliv – *Streptomyces lividans*, Mtub – *Mycobacterium tuberculosis*.

PqsD and KAS III enzymes are structurally similar, but they diverge in their biosynthesis pathways. Differences in the amino acid sequence of the active sites might account for that. A BLAST search [34] of PqsD was performed against the Uniprot and the PDB database. The closest homologues were FabH2 of several pathogenic *Burkholderia* strains (~55% sequence identity) and FabHs of Gram-positive and Gram-negative bacteria (~29-40% sequence identity). A multiple sequence alignment of PqsD with these enzymes was performed using Probcons [35] with Jalview [36] (Additional file 1: Figure. SI3-SI4). As seen in the sequence alignment all these enzymes, but in particular PqsD and *Burkholderia* FabH2, diverge in amino acid sequence in several regions (Figure 2 and Additional file 1: SI3). The domain that varies most in terms of sequence identity as well as in 3D-folding is the dimer-interface, comprising hairpin loop (**hL**; residues 185–220) and helixes H8-H9 (**h8**-**9**; residues 143–160). Further, differences are found in the “*substrate*-*loop*” (**sL**) and the adenosine binding domain (**aBS**).

As evident in PqsD and FabHs 3D-structures the **sL**s border the active site and contribute in the formation of a central cavity in the dimer structures, which is surmounted by the **hL** helixes H11 and which can be filled by water molecules (Figure 2 and Additional file 1: SI1B). Mutagenesis studies in FabH showed that residues placed within this **sL** play an exceptional role for the substrate specificity [37]: bulky residues (Phe87; *E*. *coli* FabH - *ec*FabH) determine a clear preference for the short-chained acetyl-CoA, smaller residues (Thr87; *M*. *tuberculosis* FabH - *mt*FabH) account for a long-chained substrate preference, such as lauroyl-CoA [37-40]. PqsD and *Burkholderia* FabH2 both produce HHQ and both share the same palindromic sequence (−S_82_PDHHDPS_89_-) in the **sL**. According to the sequence alignment for PqsD and FabH2 we identified Asp87 as positional analogue of *ec*FabH Phe87 and *mt*FabH Thr87 (Figure 2); consequently, also, Asp87 might be involved in the substrate recognition process.

Mutagenesis studies on *ec*FabH showed that exchanging basic residues surrounding the primary funnel access to acid residues strongly affected CoA- and ACP-binding [41]. As shown in Figure 2 in PqsD the corresponding residues are negatively charged or neutral (i.e. Glu227, Glu269, Gln270; violet triangle), which is reflected on a tertiary structure level in a modified electrostatic potential surface (Additional file 1: Figure SI5). The access to the primary funnel of PqsD is surrounded by basic residues, in part not present in other FabHs, forming a large “cationic belt” (i.e. Arg36, Arg151, Arg153, Arg221, Arg223, Arg262). This basic network forms part of the CoA and ACP binding site [31].

### Molecular dynamics simulations (A-F) of the main steps of the kinetic cycle

MD simulations of nine PqsD complexes **A**-**F** were performed to elucidate the dynamic motions within the HHQ kinetic cycle and the binding modes of ACoA and βK. The volume variations of the primary funnels, secondary channels and central cavity were tracked using the software fpocket2 [42] (Table 1). Finally, binding free energy differences (ΔG_bind_) for ACoA, βK and HHQ were estimated following a single-trajectory approach using MM-GBSA methodology (only enthalpy was computed; Table 2) and performed an MM-GBSA binding free energy decomposition analysis to highlight relevant residues for ligand binding. As two binding sites exist in the PqsD dimer we also inquired, whether synergistic effects resulted for ΔG_bind_ comparing the simulations with two ACoAs (**C2**) and βKs (**E2b**) to their single ligand counterparts (**C1** and **E2a** respectively).

**Table 1:** Internal pockets volume variations

**MD code**	**Central cavity**		**Primary funnel(chain A)**		**Primary funnel(chain B)**		**Secondary channel(chain A)**		**Secondary channel(chain B)**	
**B**	375-250	<	400-350	≤	475-500	≥	250	=	325-125	<
**C1**	375-500	>	400-530	>	450	=	300	=	400-150	<
**C2**	375	=	350-400	≥	475	=	250-300	≥	300-400	>
**D**	425-350	<	350-400	≥	375-300	<	200-350	>	400	=
**E1**	475-400	<	400-325	<	500-675	>	300	=	400-275	<
**E2a**	375-400	≥	400-525	>	500-300	<	300	=	400	=
**E2b**	300-400	>	400-450	≥	450-500	≥	250-300	≥	275-375	>
**F**	400	=	425-300	<	450-525	>	200-150	≤	275-300	≥

Summary of the volume profiles (in Å^3^) over the time for central cavity, primary funnels and secondary channels of chain A and B for each simulation **B**-**F** tracked with fpocket2. The trend progression of the volume is summarized as follows: decrease (<), modest decrease (≤), constant (=), modest increase (≥), increase (>). The time-dependent volume-profiles are added in supplementary information as Figure. SI10.

**MD codes**: **B** – PqsD apoform, **C1** – PqsD-ACoA in chain B, **C2** – PqsD with ACoA in the primary funnel of chain A and B, **D** – PqsD with Cys_112_-bound anthranilate (PqsD-CSJ), **E1** - PqsD-CSJ with βK in the primary funnel of chain B, **E2a** - PqsD-CSJ with βK in the secondary channel of chain A, **E2b** - PqsD-CSJ with βK in the secondary funnel of chain A and B, **F** – PqsD with HHQ in the primary funnel of chain B.

**Table 2:** **Estimated binding free energies** (Δ**G**_**bind**_) **using MM**-**GB**/**PBSA methods**

**MD code**	**GBSA**			
	**E**_**GAS**	**EGB**	Δ**G**_**bind**_	**STD**
**C1**	**PqsD+ACoA(chain B)**			
	−308	196	**−111**	14
**C2**	**PqsD+ACoA(chain A)**			
	−32	−50	**−82**	8
	**PqsD+ACoA(chain B)**			
	25	−88	**−63**	7
**E1**	**PqsD-CSJ+singleβK**, **primary funnel(chain A)**			
	−44	17	**−27**	2.5
**E2a**	**PqsD-CSJ+singleβK**, **secondary channel(chain A)**			
	15	−42	**−27**	6
**E2b**	**PqsD-CSJ+βK in secondary channel(chain A)**			
	−7	−37	**−44**	5
	**PqsD-CSJ+βK in secondary channel(chain B)**			
	6	−32	**−26**	6
**F**	**PqsD+HHQ (chain B)**			
	−54	14	−**40**	3

STD. - standard deviation; E_GAS - binding energy in vacuum, EGB - binding energy in implicit solvent computed with GB method.

ΔG_bind_ (kcal/mol) are computed for ACoA, βK and HHQ in the different PqsD complexes describing the kinetic cycle: **C1** – PqsD with ACoA in chain B, **C2** – PqsD with ACoA in the primary funnel of chain A and B, **D** – PqsD with Cys_112_-bound anthranilate (PqsD-CSJ), **E1** - PqsD-CSJ with βK in the primary funnel of chain B, **E2a** - PqsD-CSJ with βK in the secondary channel of chain A, **E2b** - PqsD-CSJ with βK in the secondary funnel of chain A and B, **F** – PqsD with HHQ in the primary funnel of chain B.

**Figure 3:**
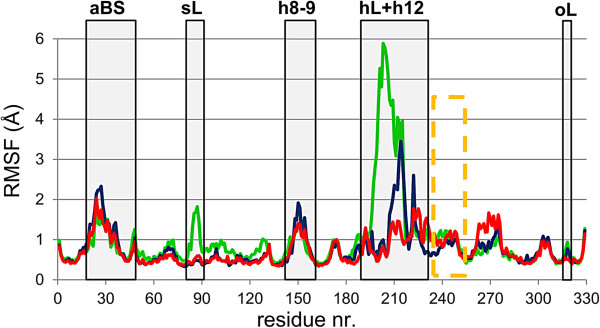
**Residue**-**dependent RMS fluctuations of the apoform MD simulations with PqsD in its monomeric and dimer state.** The fluctuations of the residues of PqsD in its monomer form (MD code **A**) are coloured in green, whereas for the PqsD in its dimer state (MD code **B**) chain A and B are shown in blue and red respectively. Domains putatively involved in the catalysis as identified by comparative analysis with homologous KAS-III enzymes are labelled with their abbreviations (dotted orange box indicates helix H14): adenosine binding site - **aBS**, palindromic “substrate-loop” - **sL**, helix H8-H9 loop - **h8**-**9**, hairpin-loop - **hL**, helix H12 - **h12**, “oxyanion-loop” – **oL**. Notably, also for all other MD simulations the largest RMS fluctuations were found in these areas (see also Additional file 1: Figure SI8).

*Flexibility of PqsD as monomer or dimer*. We investigated the flexibility of PqsD submitting monomer A of the apoform structure 3H76 to the HingeProt [43] and the HingeMaster [44] web-servers. Both predictions indicate the existence of hinges at the N- (G185/T195) and the C-terminal end (M220/G222) of the **hL** capable of large conformational changes (Additional file 1: Figure SI6A-C; morphing the transition from closed to open **hL** conformer using the Yale Morph Server [45]; Additional file 2: Movie S1: video representing hinge opening and closing). A more realistic measure of the PqsD flexibility is obtained from the monomer (**A**) and, especially, the dimer PqsD apoform MD simulations (**B**): in the monomer MD very large fluctuations are observed for the **hL** (Figure 3; green line), which collapses closing the secondary channel (Additional file 1: Figure SI7A). In the dimer MD, on contrary, **hL** and **sL** of both chains sustain each other with only the C-terminal β-sheet of the **hL** moving out thus exposing the secondary channel access. The hinge predictions and the motions seen for these MD simulations evidence the flexible nature of the **hL** and suggest an important role for it in PqsD functionality.

Accounting for all the MD simulations (**B**-**F**) a rather broad conformational ensemble was gathered. Thereby, most of the fluctuations were located in the upper third of PqsD (Additional file 1: Figure SI7B) in regions also found disordered in several FabH crystal structures. This implies that conformational rearrangements in any of them can significantly impact on the functioning of the enzymes. The most flexible domain in all the MD simulations is the dimer interface including **hL** and **h8**-**9** (Additional file 1: Figure SI8).

**Figure 4:**
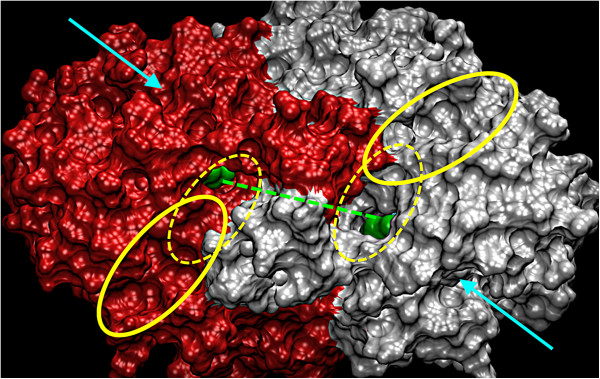
**Conformational changes at dimer interface in the dual-βK MD simulation E2b.** Top view of the dimer interface at 30 ns with the secondary channel solvent-exposed (dotted yellow eclipse) and the βK molecules shown as green surfaces. The dashed green line indicates a cleft which exists between the two H11 helixes at 0 ns, but that disappears over the simulation run. The full yellow circles indicate additional grooves formed at the interface. The cyan arrows indicate the primary funnel accesses for both chains.

The refolding of the two **hL** helixes H11 and of **h8**-**9** affected the dimer interface morphology (yellow dotted circle – cavities at 0 ns, yellow full circle – cavities at 30 ns; Figure 4) and the geometry of the arginines surrounding the primary funnel access (“cationic belt”; Additional file 1: Figure SI9). Thereby, deep channels are formed towards the central cavity (for apoform **B**, single-ACoA **C1** and the CSJ-PqsD **D** simulations) or opening out into the secondary channel (for simulations with βK in the secondary channel **E2a**-**E2b**; Figure 4).

In the apoform trajectory **B** the enzyme floats between different conformers. On contrary, in presence of the different substrates the equilibrium is selectively shifted toward one preferred structure. This is reflected in the volume variations of internal cavities and channels (Table 1 and Additional file 1: Figure SI10). In particular, tracking the central cavity volume of the MD simulations **B**-**F** resulted in a sinusoidal, “heart-beat”-like volume-profile (Table 1): in the apoform simulation (**B**) the volume is reduced; in the single-ACoA complex (**C1**) the cavity volume increases; with two ACoA molecules (**C2**) as well as for the CSJ-PqsD (**D**) complex the cavity volume decreases. Finally, in the single-βK **E2a** and, in particular, in the dual-βK simulations **E2b** the volume increased. The formation of channels from the central cavity into the oxyanion site (close to Asp87 and Ser317), sideways out to the surface, or up to the dimer interface suggests that water molecules might move along these channels depending on the catalytic needs.

The fact that ligand-induced structural changes are observed in the very same regions in presence of diverse ligands makes us confident that a sufficiently large conformational ensemble has been gathered by the MD simulations to represent a good starting point for structure-based drug design and virtual screening. A good example is represented by the motions of Phe218, which is located on the C-terminal β-sheet of the **hL** of each monomer (Additional file 1: Figure SI11): in the MDs with ACoA **C1** and **C2**, with CSJ **D**, with βK in the primary funnel **E1**, and with HHQ **F** (chain B) it stays turned towards the catalytic centre occluding the access from the catalytic site to secondary channel and dimer interface. On contrary, when βK is in the secondary channel (**E2b** and chain A of **E2a**) Phe218 rotated outwards increasing the distance between the centre of mass of Phe218 and the C_α_ of Cys112 (Additional file 1: Figure SI11); the secondary channel opened out to the dimer interface and the catalytic centre was enlarged. When no ligand is present in the catalytic centre (e.g. in the apoform MD **B** and in chain A of **F**), an intermediate position can be observed indicative for the conformational flexibility of this area.

### ACoA progression into the catalytic site

**Figure 5:**
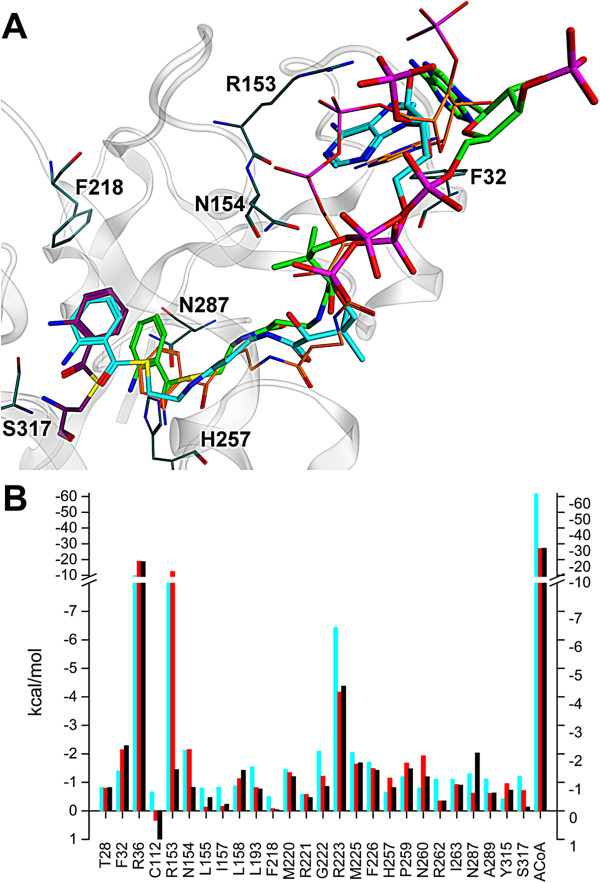
**Binding mode of ACoA. A)** Final poses of the single-ACoA simulation **C1** (cyan; 37 ns) and of the dual-ACoA simulation **C2** (green; 31 ns) are superimposed with the PDB structure 3H77 (ACoA orange - starting position; anthranilate-Cys_112_ in magenta – overlaps with ACoA_**C1**_). Phe32, Arg153, Asn154, Phe218, His257, Asn287 and Ser317 of the single-ACoA simulation at 37 ns are shown as dark cyan sticks. **B)** Decomposed energy contributions per residue (at least for one MD >0.5 kcal/mol) determined by MM-GBSA methods for the MD simulations **C1** (cyan) and **C2** (chain A – red; chain B – black).

The simulations with ACoA supplemented well the PqsD crystallographic data. In particular for the single-ACoA simulations **C1** ACoA moved deep into the primary funnel ending in a pose where the anthranilic ring overlaps well with the Cys_112_-bound anthranilate of the PDB-ID 3H77 structure (Figure 5 and SI12). Strikingly, only Cys112, His257, and Asn287 of the monomer hosting ACoA (chain B) assumed an ideal geometry for catalysis (e.g. Cys112-His257 ~4 Å; initial/final distances in Additional file 1: Figure SI12B). Arg36, Arg153, and Arg223 showed the highest binding energy contributions due to interactions with the phosphate groups of the pantetheine-linker. As obvious from the decomposed energy contributions (Figure 5B) also Thr28, Phe32, Cys112, Asn154, Leu155, Ile157, Leu158, Leu193, Phe218, Met220, Gly222, Met225, Phe226, His257, Pro259, Asn260, Ile263, Asn287, Ala289, Tyr315, and Ser317 contribute to ACoA binding. The optimal position reached by ACoA in the simulation **C1** is also reflected in a more favourable estimated ΔG_bind_ (−111 kcal/mol; chain B) than for the two ACoA molecules of the dual-ACoA simulation **C2** (−82 kcal/mol, chain A; -63 kcal/mol, chain B). Strikingly, plotting ΔG_bind_-versus-time shows a progressively more favourable ΔG_bind_ for **C1** (Additional file 1: Figure SI13), whereas for the two ACoAs in **C2** the ΔG_bind_ remain rather constant indicative for an equilibrated system, in which, however, the ACoAs in **C2** do not reach a catalysis-like position (see distances in Additional file 1: Figure SI12C). This hints at a sequential ACoA entrance/release mechanism, where the empty monomer refolds while binding of the first ACoA.

### Where and how does βK bind in PqsD?

Our SPR experiments showed that using free βK acid as second substrate yields higher HHQ formation than when it is added first. The active site of PqsD PDB structures seems inadequate to handle βK-(ACP) binding and βK-incorporation [23]. However, in PqsD a secondary channel exists similar to that of *mtFabH*, which can host long-chained β-keto-acids (i.e. lauroyl-CoA) [40]. The access to this channel is lined by polar residues (Arg145, Thr195, Ser317, and Asp87 of the second monomer), whereas its bottom part is rather hydrophobic (Leu81, Leu142, Leu155, Leu159, Leu193, Met194, Phe218 and Met220). Further, this secondary channel borders the central cavity and the dimer interface with the ion-pair Asp87-Arg145 (of the other chain) and Phe218 acting as barrier respectively. As PqsD is clearly capable of HHQ biosynthesis conformational changes are expected, in analogy to *mtFabH*[46], that allow HHQ formation and release.

We followed two approaches to determine the most plausible access path and binding mode of βK. In the first, we docked βK into the primary funnel of anthranilate-ligated PqsD. Two main orientations resulted: 1) with the polar head of βK pointing to the catalytic triad (Additional file 1: Figure SI14A); and 2) turned 180° with the carboxylic group interacting with the Arg of the cationic belt. The second binding mode was not supportive for any of the catalytic mechanisms [10,17].

The second approach was based on the substrate size similarity and the structural homology between PqsD and *mt*FabH. We superimposed the Cys112-anthranilate PqsD (PDB-ID 3H77) and *mtFabH* co-crystallized with dodecyl-CoA (PDB-ID 1U6S) [46] and then transposed and modelled the 3-oxo-undecanoyl from the *mtFabH* structure into βK in the secondary channel of PqsD (Additional file 1: Figure SI14B). Active site refinement with the LigX module of MOE [47] with restrained βK resulted in an energy optimized complex with small C_α_ RMSD (~ 1 Å) compared to the starting complex. This second binding mode of βK looks similar to that obtained by Bera et al. [23] for decanethiol by superimposing PqsD with the *mt*FabH structure PDB-ID 2QO1 where the decanethiol is covalently attached to the catalytic Cys.

The MD simulations **E1**-**E2b** with βK either in the primary or in the secondary channels were supportive to understand the putative ping-pong kinetic cycle.

*MD simulation CSJ*-*PqsD with* β*K in the primary funnel of chain B* (**E1**; 34 ns): Only small conformational changes are observed in this MD simulation for βK, which points the 3-oxo-β-keto moiety towards the catalytic triad (Additional file 1: Figure SI15). Thereby, the carboxylate head is trapped in a hydrogen-bond network with His257 and Asn287 holding the β carbon of βK close to the CSJ-sulphur as shown by their favourable energy contributions (Additional file 1: Figure SI15B). Additionally, van der Waals interactions are formed between βK and Leu193, Met220, Met225, Phe226, Pro259, Ile263, and Tyr315 (Additional file 1: Figure SI15).

**Figure 6:**
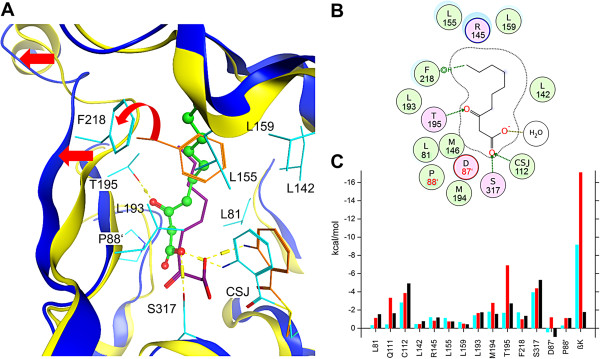
**Binding mode of βK in the secondary channel (MD simulations E2a and E2b). A)** Superimposed snapshots of βK binding to PqsD at 0 and 30 ns. βK at 0 and 30 ns is shown as purple sticks and as green ball and sticks respectively. Important residues are shown as cyan lines at 30 ns and as orange lines at 0 ns. The red arrows indicate the conformational shift from the initial (yellow) to the final (blue) conformation of PqsD in the MD simulation **E2b**; Phe218 flips out in presence of βK. **B)** Schematic representation of βK in the secondary channel. Polar amino acids are illustrated in purple and hydrophobic amino acids in green circles. Hydrogen bonds and CH-pi interactions are shown as green arrows and dotted lines. **C)** Decomposed energy contributions per residue (at least for one MD >0.5 kcal/mol) determined by MM-GBSA methods for the MD simulations **E2a** (cyan) and **E2b** (chain A – red; chain B – black).

*MD simulation CSJ*-*PqsD with βK in the secondary channel of chain A* (**E2a**; 30 ns): After 30 ns the carboxylic moiety of βK protruded into the catalytic centre within 4.5 Å of the amine of CSJ, the hydroxy group of Ser317 and of the oxyanion site formed by Gln111 and Ser317. Further, βK forms a hydrogen bond with Thr195 as shown in Figure 6. Phe218 was pushed outwards by βK in chain A (Figure 6A and Additional file 1: Figure SI11), while in the empty chain B Phe218 points towards the catalytic centre (Additional file 1: Figure SI11), reducing the primary funnel volume of chain B (Additional file 1: Figure SI10).

**Figure 7:**
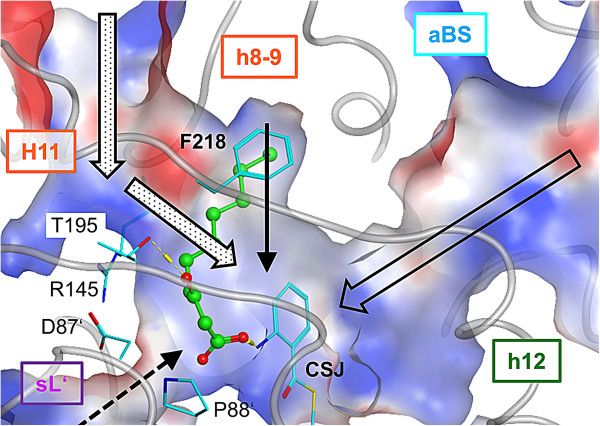
**U-shaped channel network formed in presence of βK in the secondary channel.** The U-shaped channel connects primary funnel access with the catalytic centre (large unfilled arrow), the secondary channel (thin arrow) and the dimer interface (large dotted arrows). In presence of βK a tunnel is formed between Cys_112_-bound anthranilate (CSJ) and the central cavity (dashed thin arrow) potentially used for water supply (needed for catalysis) from the central cavity to the oxyanion site close to P88′. βK is shown as green ball and sticks, whereas residues shown to interact with βK (CSJ, T195, F218, R145, D87′, P88′) as cyan sticks. For clarity the domains are labelled with their abbreviations. The solvent accessible surface is color-coded as follows: blue – positive electrostatic potential (+25 kcal/mol); red – negative electrostatic potential (−25 kcal/mol), white – neutral.

*MD simulation CSJ*-*PqsD with* β*K in both secondary channels* (**E2b**; 30 ns): As seen comparing the single- and the dual-ACoA MD simulations **C1** and **C2** (Additional file 1: Figure SI17 and SI18) simulating two βK molecules simultaneously resulted in a more stable trajectory (smaller RMSD as for **E2a**; Additional file 1: Figure SI21 and SI22). Still, the modelled presence of βKs in the secondary channels induce structural changes in the dimer-interface and in neighbouring domains (**hL**, **h8**-**9**, **h12** and **oL** of one monomer and **sL** of the other monomer; Additional file 1: Figure SI22). Two long U-shaped tunnels are formed, which connect primary funnel access, catalytic site (with CSJ and βK in a productive pose) and secondary channel access at the dimer interface (Figure 7). The two βK are in a similar or even more optimal pose (see βK in chain A) as described for the single-βK MD **E2a**. Phe218 is turned outside (Figure 6A and SI11) exposing the secondary channels of chain A and B to the dimer-interface (Figure 4). Hydrogen bonds are formed between the carboxylic moiety of βK and the amine of CSJ and the hydroxy group of Ser317. Further, a hydrogen bond is formed between the β-keto carbonyl and Thr195 holding βK close to the “substrate-loop” (**sL**) and in particular to Pro88 and Asp87 (see binding energy contributions; Figure 6C). Finally, Van der Waals interactions are established between βK and Leu81, Pro88, Leu155, Leu159, Leu193, Met194 and Phe218.

### Catalytic mechanism and βK binding modes

In this study the free acid form of βK was used to test HHQ biosynthesis. Two aspects have thus to be considered: firstly, the free βK-acid is unlikely to exist in bacteria and its *K*_D_ value in the millimolar range determined by SPR and enzyme kinetics does not fit with the substantial HHQ production in *P*. *aeruginosa*. Secondly, inverting the substrate order in either the SPR or the enzyme kinetic studies still yields HHQ formation, although substantially decreased (see Additional file 1: Figure SI2A).

**Figure 8:**
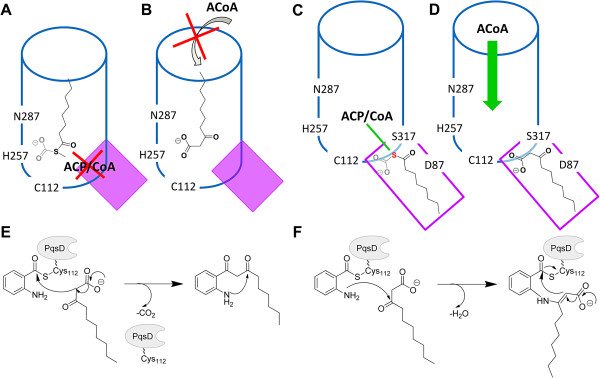
**Binding modes of βK and putative catalytic mechanisms of βK-incorporation. A)** βK is placed in the primary funnel (blue tube) as in the MD simulation **E1**: no space is left to host the thioester carrier CoA/ACP. **B)** The access of ACoA into the primary funnel is hindered with βK in the pose as in A. **C)** βK is in the secondary channel (violet box) as in the MD simulations **E2a** and **E2b**: in the primary funnel space is available to accommodate the thioester carrier CoA/ACP. **D)** ACoA can access the primary funnel with βK in this pose and transfer anthranilate to Cys112. **E)** The Claisen condensation fits best as mechanism for βK incorporation for βK in the primary funnel. **F)** The imine/enamine formation is the most plausible mechanism for βK incorporation when βK is in the secondary channel.

The final pose identified in the simulation **E1** with βK in the primary funnel hints at a potential βK-activation via decarboxylation, which could occur either spontaneously or facilitated by His257 and Asn287 in analogy to other KAS-III enzymes.[24] This pose of βK fits well with the decarboxylative Claisen reaction (Figure 8E). However, both of the above-mentioned aspects argue against this binding mode as the “*in vivo*” one: the orientation of βK cannot match that of any thioester-bound form needed for its delivery (Figure 8A), and, in addition, no access to the catalytic site for ACoA as second substrate is possible as long as βK is placed in the primary channel as seen for **E1** (Figure 8B).

Given the unlikeliness of free βK acid as “*in vivo*” substrate other events must occur in HHQ biosynthesis, such as involvement of PqsB and PqsC [31] or ACP-thioester binding, which could reduce the activation energy or favour the kinetics. Holding true the ACP-delivered βK, we notice that the binding site topology and final orientation of βK in the secondary channel (MDs **E2a**-**E2b**) does not disturb the access of ACoA (Figure 8C) nor it precludes thioester-delivered βK (Figure 8D). The interactions with CSJ112, Thr195, and Ser317 found for **E2a**-**E2b** (see energy contributions in Figure 6C) fit well with the imine/enamine mechanism proposed by Diggle et al. [10] (Figure 8F). An equal or even more favourable ΔG_bind_ is estimated for the two βK in the dual-βK simulation **E2b** (−44 kcal/mol – chain A, -26 kcal/mol – chain B) as compared to ΔG_bind_ of the single-βK (−27 kcal/mol in **E1**; -27 kcal/mol in **E2a**) (Table 2). This hints at a positive synergistic effect of the simultaneous presence of two βK molecules in the secondary channels, which might facilitate conformational changes necessary to accommodate βK in an energetically favoured pose. In this regard, it is helpful to remember that in the dual-βK simulation a dynamic channel-system is formed, which enlarges the catalytic centre (Additional file 1: Figure SI10), thus accounting for a putative intramolecular cyclization in HHQ formation. Although no conclusive data exists all the above aspects let us favour the secondary channel binding mode as the more probable.

### Ser317Phe PqsD mutant

In the MD simulations Ser317 is involved in hydrogen bonds with the carbonyl-group of ACoA (**C1** and **C2**) and with the carbonyl- or the carboxylic-moiety of βK (**E2a** and **E2b**). To verify its importance we exchanged Ser317 by site-directed mutagenesis into Phe and determined the catalytic activity by detection of the formed HHQ using UHPLC-MS/MS. Under assay conditions (0.1 μM enzyme, 5 μM ACoA, 70 μM βK) the mutant produced less factor 700 HHQ compared to the wild type. Tenfold higher enzyme concentration of the S317F did not result in an increase in HHQ production indicating a complete abolishment of the catalytic functionality.

**Figure 9:**
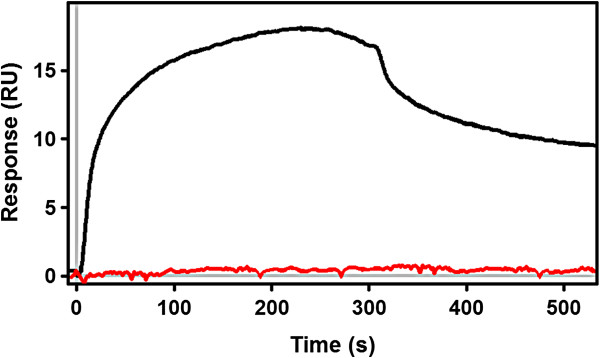
**SPR sensograms of ACoA.** When ACoA is injected with PqsD wild-type fixed on the chip (black) an increase in the response units is observed corresponding to the anthranilate transfer to Cys112. Differently, when S317F PqsD mutant (red) is present on the chip, no increase is observed.

In order to understand at which stage of the kinetics the S317F mutant disrupts the biosynthesis we performed SPR experiments using S317F PqsD. Strikingly no anthranilate transfer to Cys112 was detected (Figure 9) suggesting that substitution of Ser317 with Phe already disrupts the first step of the kinetics. The close vicinity of Ser317 to the catalytic site, however, makes it questionable whether the very weak enzyme activity is due to the rooted ability of S317F to form hydrogen bonds, as suggested by the consistent binding energy contributions of Ser317 in most of the MD simulations with both ACoA and βK (Figure 5 and 6), or because of the primary funnel being occluded by the newly introduced aromatic ring.

## Conclusions

In this work we elucidated some of the molecular bases of HHQ biosynthesis. A putative ping-pong kinetic mechanism was determined by enzyme kinetics experiments, which was substantiated by the preferred substrate order (ACoA prior to βK) assessed by SPR. These data were useful to set-up and analyse the MD simulations, which aimed to unmask dynamic motions governing PqsD functionality. MD simulations reveal a kinetic-step dependent adaptation of PqsD to the diverse ligands, a favoured binding mode for βK in the secondary channel, as well as an arginine-assisted progression of ACoA towards the catalytic site. Also, Ser317 was identified as an important binder for ACoA and βK, at least in part confirmed by the inactive S317F PqsD mutant.

In general, SPR studies with PqsD mutants will be helpful to determine the binding mode of inhibitors. Finally, the conformational ensemble retained from the MD simulations will serve as valuable starting point for structure-based design of PqsD inhibitors.

## Methods

### *Production and purification of recombinant PqsD in* E. coli

The overexpression and purification of PqsD as an N-terminal His_6_-tagged fusion protein in *E*. *coli* BL21 (ʎDE3) using the vector pET28b(+) -*pqsD* was performed as described by Pistorius et al. [17]. To remove the His_6_-tag the protein was subjected to thrombin cleavage performed at 16°C for 16 h in a 50 mM Tris–HCl buffer, pH 8.0, containing 150 mM NaCl, 1 mM 2-ME, 2.5 mM CaCl_2_ and 1 unit thrombin per mg protein followed by a second passage through the His Trap HP 5 column. The protein was frozen in liquid nitrogen.

### Preparation of S317F PqsD mutant

S317F PqsD mutant was generated using the QuikChange Site-Directed Mutagenesis Kit (Stratagene, La Jolla, CA) according to the manufacturer’s instructions using the pET28b(+)/*pqsD* plasmid as template and the mutagenesis primers 5′ GCTGGTCCTGACCTACGGTTTTGGCGCGACCTGGGGCG 3′ and 5′CGCCCCAGGTCGCGCCAAAACCGTAGGTCAGGACCAGC 3′. Plasmid DNA was purified using the GenEluteTM HP Plasmid Miniprep Kit (Sigma-Aldrich, St. Louis, MO) and sequenced to confirm the site-directed mutation.

### Enzyme kinetic analysis

The PqsD catalysed formation of HHQ was analysed using a UHPLC-MS/MS based assay performed in 96-well microtiter plates (Greiner) using the method of Pistorius et al. [17] with some modifications. First, the purified enzyme PqsD (0.8 μM; in 50 mM MOPS, pH 7.0, 0.016% (v/v) Triton X-100) was preincubated without substrates for 5 min at 37°C. The reaction buffer (15 μL; 50 mM MOPS, pH 7.0) and the substrates ACoA (20 μL; concentrations: 2–40 μM) and ßK (20 μL; concentrations: 240–4000 μM) were added. The reaction was started by the addition of preincubated enzyme (25 μL; 0.8 μM) resulting in a total reaction volume of 80 μL with the following final concentrations: PqsD 0.25 μM, ACoA 0.5 - 10.0 μM and ßK 60–1000 μM, Triton X-100 0.005%, methanol 2%. The reaction was stopped after 4 min at 37°C by adding 80 μL of methanol containing 1 μM of the internal standard amitriptyline. For each sample, the reactions were performed in quadruplicate. HHQ-formation was detected using UHPLC-MS/MS according to method in supplementary information (Additional file 1: Text SI1). Data were plotted and analysed using GraphPad Prism 5 software.

*Synthesis of Anthraniloyl*-*S*-*Coenzyme A thioester* (*ACoA*). ACoA was synthesized from isatoic anhydride and coenzyme A (CoA) as described by Simon and Shemin [48].

*Synthesis of ethyl 3*-*oxodecanoate*, *3*-*oxodecanoic acid* (β *ketodecanoic acid*), *and of HHQ* (*2*-*heptylquinolin*-*4*(*1H*)-*one*). Synthesis as described by Lu et al. [49].

### Surface Plasmon resonance (SPR)

SPR binding studies were performed using a Reichert SR7000DC optical biosensors instrument (Reichert Technologie, Depew, NY 14043 USA). HC1000m sensor chips were purchased from Xantec Analytics (Düsseldorf, Germany).

#### Immobilization of H_6_PqsD or H_6_PqsD S317F

Overexpression and purification of PqsD or of the S317F PqsD mutant was performed as previously described [32]. *H*_*6*_*PqsD* or *H*_*6*_*PqsD S317F* was immobilized on HC1000m sensor chip using standard amine coupling chemistry at 25°C analogous to the manufacturer’s instruction. *H*_*6*_*PqsD or H*_*6*_*PqsD S317F* was diluted into 10 mM sodium acetate (pH 4.5) to a concentration of 100 μg/mL and coupled to the surfaces with densities between 15000 and 20000 RU.

#### Catalytic activity of PqsD

ACoA was diluted into running buffer to 10 μM. β-ketodecanoate (βK) was dissolved in methanol to a 10 mM stock solution and diluted into running buffer to 20 μM. In the first experiment ACoA was injected for 5 min association and 10 min dissociation time, followed by a 20 min injection of βK. In the second experiment βK was added to the running buffer (100 μM in 50 mM Tris, pH 8.0, 150 mM NaCl, 0.1% Triton X-100). The ACoA-injection (10 μM for 5 min) followed when the binding site was saturated, indicated by a stable baseline. In all experiments the flow through was collected. After ACoA addition CoA emission was detected using UHPLC-MS/MS as reported elsewhere [32]. After addition of the second substrate (experiment dependent) the flow through was extracted with 1 mL CHCl_3_, evaporated and diluted in 50 μL MeOH. HHQ formation was detected using UHPLC-MS/MS (see Additional file 1: Text SI1).

#### SPR study with S317F PqsD mutant

For comparison of ACoA binding to PqsD wild- type and to S317F PqsD mutant, the study was performed as in the first experiment described above.

### Computational methods

*Docking of* β*K and HHQ*. βK and HHQ were docked each 50 times with GOLDv5.0 [50] using the GOLDSCORE function [51]. The docking site was defined including all residues within 9 Å of ACoA found in PDB structure 3H77. Covalent ligated PqsD and apoform PqsD were used to dock βK and HHQ, respectively. The default GOLD parameters were used.

#### Simulation protocol

System setup was performed as follows for all simulated systems. Atomic coordinates were taken from PDB-ID 3H76 for MD simulations **A**, **B**, **C1**, **C2** and **F**, and from 3H77 for **D**, **E1**, **E2a** and **E2b**. Water molecules and ions present in the crystals were removed. The protonation states were determined at pH 7.4 with the Protonate3D module of MOE and the enzyme complexes then minimized using the MOE module ligX [47]. The solvated systems were set up using the AMBER11 [52] program xLeap with AMBER99SB force field [53]. A 9 Å pad of TIP3P waters was added to solvate each system as octahedral box. Neutralizing counter ions were added to each system.

Parameters for ACoA, βK, HHQ and the Cys112-bound anthranilate (CSJ) were determined using the sqm routine of AmberTools1.5 [52]. For each ligand AM1-BCC charges were computed. For ACoA and βK a net charge of -3 and -1 was set, respectively. For CSJ, which was taken from the PDB-ID 3H77, the Cys parameters were used as starting point followed by parameterization with the sqm routine.

MD simulations were performed with the parallelized PMEMD module of AMBER 11. The starting PqsD complexes were minimized and equilibrated with the backbone atoms restricted by harmonic restraints of initially 10 kcal mol ^−1^ Å^–2^ and then progressively reduced to 5, 2, 1 and 0 kcal/mol. The systems were heated to 300 K in the canonical NVT ensemble (constant number of particles, N; constant volume, V; constant temperature, T) using a Langevin thermostat, with a collision frequency of 3.0 ps^−1^ Å^−2^. Production runs were then made for 30–37 ns duration in the NPT ensemble at 300 K. As with the heating, the temperature was controlled with a Langevin thermostat (but with a 1.0 ps^−1^ collision frequency). The time step used for all stages was 2 fs and all hydrogen atoms were constrained using the SHAKE algorithm [54]. Long-range electrostatics were included on every step using the Particle Mesh Ewald algorithm with a 4th order B-spline interpolation [55].

#### MD analysis

B-factor, distances and RMSD time series were calculated using the cpptraj analysis tool of the AmberTools 1.5 package.

Structures were sampled at 20 ps intervals. RMSD values were calculated for the enzyme, but also for each of the following regions individually, facilitating the interpretation of the data: adenosine binding site (**aBS**), “substrate-loop” (**sL**), helix8-9 (**h8**-**9**), hairpin loop (**hL**), helix 12 (**h12**), oxyanion loop (**oL**).

#### Volume variations of internal cavities

The volume variations (Å) over the time (ns) for five cavities in PqsD were tracked for all PqsD dimer MD simulations using the software package fpocket2 [42]. All MD snapshots were superimposed using the C_α_ of the apoform PDB-ID 3H76 as reference structure. Default parameters for the identification of small cavities and channels were used. All plots are added in supplementary materials.

#### MM-GBSA calculations

Binding energies ΔG_bind_ for ACoA, βK and HHQ were estimated by conventional MM-GBSA methods (Molecular Mechanic – General Born Surface Area) [56] using snapshots of the simulations sampled every 30 ps. Energy decomposition analyses with Generalized Born solvent were performed with per-residue decomposition and 1–4 interactions added to the electrostatic and Van der Waals terms (idecomp = 2). More details to the MM-GBSA method are in Additional file 1: Text_SI2.

A detailed description of the MD simulations **A**-**F**, cavity volume variation-*versus*-time profiles (Additional file 1: Figure SI7), time-dependent ΔG_bind_ profiles (Additional file 1: Figure SI10), and RMSD plots (Additional file 1: Figure SI13-SI20) are added as additional data. The RMSD analyses of the different regions helped to visualize motions in the MD simulations not visible from the all-atom RMSD plots for the entire protein and complemented the amino acid fluctuation analyses shown in Figure 3.

#### Figures and plots

Plots were made with Origin 9 or Excel, while figures with PyMOL [57] or MOE [47].

## Abbreviations

QS: quorum sensing; PQS: Pseudomonas Quinolone Signal; MD(s) simulations: Molecular Dynamics simulations; SPR: surface plasmon resonance; HHQ: 2-heptyl-4-hydroxyquinoline; HAQ: 2-alkyl-4-hydroxyquinoline; ACoA: Anthranoyl-CoA; βK: β-decanoate; CSJ: Cys112-anthranilate; ACP: acyl carrier protein; ecFabH: *E*. *coli* FabH; mtFabH: *M*. *tuberculosis* FabH.

## Competing interests

The authors declare that they have no competing interests.

## Authors’ contributions

AS participated in the design of the study and drafted the manuscript. CKM performed the enzyme kinetic analysis and helped to draft the manuscript. LW and CH participated in the design of the study and performed the SPR experiments; CB carried out the mutagenesis experiments. RWH conceived of the study, and participated in its design and coordination. MN performed sequence alignment, docking studies, molecular dynamics simulations, conceived of the study and drafted the manuscript. All authors read and approved the final manuscript.

## Supplementary Material

Additional file 1 **Supplemental methods, figures and references.** The file is in PDF format. It includes: **Text SI1** - UHPLC-MS/MS analysis of HHQ. **Text SI2** - MM-GBSA theory. **Text SI3** - Description of molecular dynamics simulations A-F. Supplementary Information Figures. **Figure SI1** - Structural peculiarities of PqsD. **Figure SI2** - HHQ biosynthesis follows a ping-pong mechanism. **Figure SI3** - Multiple sequence alignment of PqsD and KAS III enzymes. **Figure SI4** - Average distance tree from the ProconsWS alignment. **Figure SI5** - Electrostatic potential of PqsD (A) and *E*. *coli* FabH (B). **Figure SI6** - Predicted hinge regions in PqsD. **Figure SI7** - PqsD flexibility in the single monomer MD simulations A. **Figure SI8** - Residue-dependent RMS fluctuations for the MD simulations A-F. **Figure SI9** - Conformational changes of the cationic belt. **Figure SI10** - Time-dependent volume variations of internal cavities. **Figure SI11** - Time-dependent distance variation between Phe218 and Cys112. **Figure SI12** - Progression of ACoA in the single-ACoA MD simulation C1. **Figure SI13** - Time-dependent variation of the estimated binding free energy. **Figure SI14** - Where does βK bind in PqsD? **Figure SI15** - Binding mode of βK in the MD simulation E1. **Figure SI16**-**S23** - Trajectory analysis of the MD simulations B-F. Supplementary information References. Click here for file

Additional file 2: Movie S1 The morphing from the closed to the open hairpin-loop (hL) conformation is showed as result of the YaleMorphServer. The file is in avi format. Click here for file
