# Supplementary material for: Molecular basis of HHQ biosynthesis: molecular dynamics simulations, enzyme kinetic and surface plasmon resonance studies
Source: BMC Biophys. 2013 Aug 1;6:10. doi: 10.1186/2046-1682-6-10 (PMC3734052; doi:10.1186/2046-1682-6-10)
Supplement: Additional file 1 — Supplemental methods, figures and references. The file is in PDF format. It includes: Text SI1 - UHPLC-MS/MS analysis of HHQ. Text SI2 - MM-GBSA theory. Text SI3 - Description of molecular dynamics simulations A-F. Supplementary Information Figures. Figure SI1 - Structural peculiarities of PqsD. Figure SI2 - HHQ biosynthesis follows a ping-pong mechanism. Figure SI3 - Multiple sequence alignment of PqsD and KAS III enzymes. Figure SI4 - Average distance tree from the ProconsWS alignment. Figure SI5 - Electrostatic potential of PqsD (A) and E. coli FabH (B). Figure SI6 - Predicted hinge regions in PqsD. Figure SI7 - PqsD flexibility in the single monomer MD simulations A. Figure SI8 - Residue-dependent RMS fluctuations for the MD simulations A-F. Figure SI9 - Conformational changes of the cationic belt. Figure SI10 - Time-dependent volume variations of internal cavities. Figure SI11 - Time-dependent distance variation between Phe218 and Cys112. Figure SI12 - Progression of ACoA in the single-ACoA MD simulation C1. Figure SI13 - Time-dependent variation of the estimated binding free energy. Figure SI14 - Where does βK bind in PqsD? Figure SI15 - Binding mode of βK in the MD simulation E1. Figure SI16-S23 - Trajectory analysis of the MD simulations B-F. Supplementary information References. [file 2046-1682-6-10-S1.pdf]

Additional file 1 – Supplemental methods, figures and references

Molecular Basis of HHQ biosynthesis: Molecular Dynamics Simulations, Enzyme Kinetic and Surface Plasmon Resonance Studies

Anke Steinbach [1*], Christine K. Maurer [1*], Elisabeth Weidel [3], Claudia Henn [1†], Christian Brengel [1], Rolf W. Hartmann [1,2], Matthias Negri [1§]

[1] Helmholtz-Institute for Pharmaceutical Research Saarland, Campus C2.3, 66123 Saarbrücken, Germany
[2] Pharmaceutical and Medicinal Chemistry, Saarland University, Campus C2.3, 66123 Saarbrücken, Germany
[3] ElexoPharm GmbH, Im Stadtwald A1.2, 66123 Saarbrücken, Germany

*These authors contributed equally to this work

[†]Current address: MIP Pharma GmbH, Kirkelerstr. 41, 66440 Blieskastel-Niederwürzbach, Germany.

[§]Corresponding author: Matthias Negri, Department of Drug Design and Optimization, Helmholtz-Institute for Pharmaceutical Research Saarland, Campus C2.3, 66123 Saarbrücken, Germany, Tel.: +49-681-302 70328; Fax: +49-681-302 70308; Email: matthias.negri@helmholtz-hzi.de

Table of Contents

Text SI1. UHPLC-MS/MS analysis of HHQ 2
Text SI2. MM-GBSA theory 2
Text SI3. Description of molecular dynamics simulations **A**-**F** 3

Supplementary Information FIGURES
FIGURE SI1. Structural peculiarities of PqsD. 4
FIGURE SI2. HHQ biosynthesis follows a ping-pong mechanism 5
FIGURE SI3. Multiple sequence alignment of PqsD and KAS III enzymes 6
FIGURE SI4. Average distance tree from the ProconsWS alignment 7
FIGURE SI5. Electrostatic potential of PqsD (A) and *E. coli* FabH (B) 8
FIGURE SI6. Predicted hinge regions in PqsD 9
FIGURE SI7. PqsD flexibility in the single monomer MD simulations **A** 10
FIGURE SI8. Residue-dependent RMS fluctuations for the MD simulations **A**-**F** 10
FIGURE SI9. Conformational changes of the cationic belt. 11
FIGURE SI10. Time-dependent volume variations of internal cavities 12
FIGURE SI1. Time-dependent distance variation between Phe218 and Cys112 13
FIGURE SI12. Progression of ACoA in the single-ACoA MD simulation **C1** 14
FIGURE SI13. Time-dependent variation of the estimated binding free energy 15
FIGURE SI14. Where does βK bind in PqsD? 15
FIGURE SI15. Binding mode of βK in the MD simulation **E1** 16
FIGURE SI16-S23. Trajectory analysis of the MD simulations **B**-**F** 17-24

Supplementary information REFERENCES 25

Text SI1. UHPLC-MS/MS analysis of HHQ.

The analyses were performed using a TSQ Quantum Access Max mass spectrometer (Thermo Scientific Finnigan, San Jose, CA). The MS detection was carried out in heated ESI mode, at a spray voltage of 5.0 kV, a probe temperature of 350 °C, a nitrogen sheath gas pressure of 4.0×10^5 Pa, an auxiliary gas pressure of 2.0×10^5 Pa and a capillary temperature of 380 °C in positive ionization mode. Observed ions were (mother ion [m/z], product ion [m/z], scan time [s], scan width [m/z], collision energy [V], tube lens offset [V]): HHQ: 244, 159, 0.1, 0.01, 33, 88; amitriptyline (internal standard): 278, 233, 0.1, 0.01, 15, 58. The chromatographic separation was carried out on an Accela UHPLC using an Accucore RP-MS column (150 x 2.6 µm) with an injection volume of 25 µL. The solvent system consisted of 10 mM ammonium acetate and 0.1 % TFA (A) and acetonitrile containing 0.1 % TFA (B). In a gradient run the percentage B was increased from an initial concentration of 60 % to 98 % in 1.00 min, kept for 0.7 min. The injection volume was 25 µL and the flow rate was set to 880 µL/min. Xcalibur software (version 2.1.0 SP1.1160) was used for data acquisition and for the quantitative evaluation of the MS data relative to a calibration curve.

Text SI2. MM-GB/PBSA.

Conventional MM-GB/PBSA [1-2] calculations were performed using the AMBER 11 suite.[3] The electronic and Van der Waals energies were calculated by the Sander module. The binding free energy resulting from the formation of the protein-ligand complex is approximated by the following equation:

$$\Delta G = \Delta H - T\Delta S \qquad (1)$$

in which *T* is the temperature of the system at 300 Kelvin.

The binding free energy (ΔG) of the protein–ligand complex resulted from:

$$\Delta G = G_{complex} - (G_{protein} + G_{ligand}) \qquad (2)$$

where $G_{complex}$ is the absolute free energy of the complex, $G_{protein}$ is the absolute free energy of the protein, and G_{ligand} is the absolute free energy of the ligand.

For each species (complex, protein, and ligand) snapshots were extracted from all stable sectors of the trajectories lasting at least 4 ns every 30th step.

The enthalpy term in (1) consists of following subenergy terms:

$$H_{tot} = H_{gas} + G_{solv} \qquad (3)$$

$$H_{gas} = E_{elec} + E_{vdw} + E_{int} \qquad (4)$$

where H_{gas} is the potential energy of the solute, E_{vdw} the sum of van der Waals energy, E_{elec} the electrostatic energy and E_{int} the internal energies in gas phase by using the SANDER module of Amber. G_{solv} is the solvation free energy and is given by the sum of electrostatic (G_{elec}) and nonelectrostatic (hydrophobic) contributions (G_{nonel}):

$$G_{solv} = G_{elec} + G_{nonel} \qquad (5)$$

The polar solvation energy was calculated with the finite-difference PB equation solver by using AMBER toolset. A spherical solvent probe (radii) of 1.4 Å and atomic radii provided by the Amber force field were used for the implicit solvent molecules and solute atoms, respectively, during the PBSA computations. Parameter/topology files used in MM-GB/PBSA computations were prepared for the complex, the protein, and the inhibitors using the LEAP module. Snapshots extracted from trajectories were pre-minimized in the gas phase by the SANDER module using a conjugate gradient method until the root-mean-square-deviation of the elements of the gradient vector was less than 10^{-4} kcal/mol^{-1}Å^{-1}.

Text SI3. Molecular Dynamics Simulations of the single kinetic steps (A-F).

*Apoform monomer (***A***;* 30 ns): In order to verify the hinge predictions we carried out an MD simulation with monomer A of the apoform crystal. The most consistent motions affect the adenosine binding site (**aBS**), the "substrate-loop", and the hairpin-loop (**hL**), thus matching the hinge prediction. However, in contrast to the hinge prediction, the **hL** collapses towards the catalytic triad occluding the access to the secondary channel (Fig. SI7).

*Apoform dimer (****B****;* 30 ns): The trajectory is characterized by fluctuations between diverse enzyme conformers. The access to the primary funnel is enlarged by concerted rearrangements involving **aBS**, helixes H8 and H9 (**h8-9**), C-terminal β-sheet of the **hL,** and helix H12 (Fig. 3 and SI16). Phe218 can rotate outwards away from Cys112 enlarging the catalytic centre/primary funnel, but in turn shrinking the central cavity (Fig. SI10). These motions suggest that the enzyme might have to refold in order to accommodate incoming ACoA molecules.

Apoform with ACoA in chain B (**C1***;* 37 ns): The fluctuations (RMSD-amplitudes; Fig. SI17) are reduced compared to the apoform simulation: they persist for chain A, but chain B is stabilized due to the presence of ACoA (Fig. 3). Already in the first ns ACoA moves from the starting position (~ 9 Å to Cys112) to the bottom of the primary funnel (Fig. SI9). Here, the anthranilic moiety of ACoA overlaps with the Cys112-ligated anthranilate (CSJ) of 3H77 (Fig. 5): the thioester carbonyl carbon moves within 4.5 Å of the Cys112-sulphur and close to His257, Asn287 and Ser317. The anthranilic moiety interacts with Phe218 (edge-to-face stacking) and with Ser317 (hydrogen bond between amine of CSJ and OH of Ser). The distance between the two His86 of the **sL**s is increased with consequent enlargement of central cavity (Fig. SI10). The adenosine moiety slightly rearranges: while preserving the π-stacking with Phe32 and Arg153, which is however weakened (see binding energy contribution of **C1** compared to **C2** in Fig. 5B), the adenosine is flipped vertically pushing the panthetheine-arm deeper into the funnel.

Apoform with two ACoAs (**C2**; 30 ns): The presence of ACoA in both primary funnels enforces the stability of the complex (Fig. SI8 and Fig. SI18) A rapid progression of the ACoAs into the primary funnels occur, but, in contrast to what seen in **C1**, not deep enough to reach a catalysis-like position (Fig. 5; thioester carbonyls are too far from Cys112-S 7/9 Å, see distance plots in SI12).

Cys112-anthranilate PqsD (CSJ-PqsD) (**D**; 37 ns): An unexpected "double-faced" profile is observed for this simulation: stable in the first 15 ns, and strongly perturbed in the second half of the MD (Fig. SI19). The CSJs bend towards Asp87, which induces His86 of both chains to move closer with consequent restriction of the central cavity volume (Fig SI10) and refolding of "substrate"- (RMSD increases to 2Å) and "oxyanion"-loop (with Ser317). Further, the motions of the CSJs lead to an enlargement of the catalytic centre involving primary and secondary channels (Fig. SI10). As also observed for the apoform MDs without ligands in the primary funnel the adenosine binding site rearranges (Asn154 binds to Arg36): in this final conformation the CSJ-PqsD complex appears not able to accommodate the adenosine of ACoA.

Apoform with HHQ in chain B (**F**; 30 ns): The starting complex was obtained by docking HHQ into the primary funnel of the apoform structure. Our goal was to identify protein dynamics elucidating the HHQ exit-mechanism, such as e.g. hinge opening. However, HHQ remained trapped into the primary funnel with a stable RMSD profile (Fig. SI24) and a quite favourable ΔG_{bind} of -40 kcal/mol. Thus, this simulation was not helpful, suggesting that a different PqsD conformation must exist when HHQ exits.

Supplementary Information FIGURES

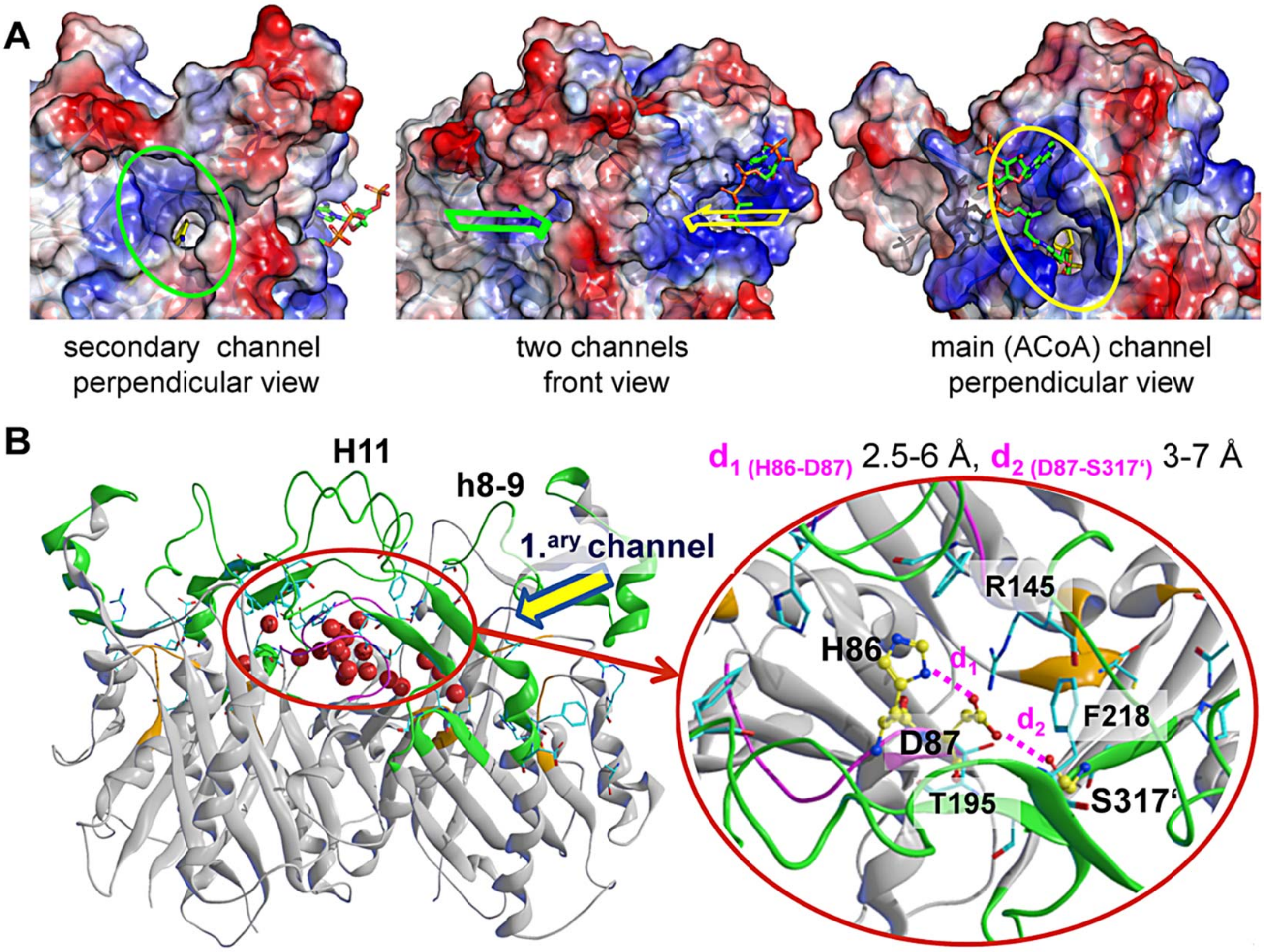

FIGURE SI1 - Structural peculiarities of PqsD. A) Electrostatic surface of a single chain of PqsD shown as front view (primary and secondary channel indicated by green and yellow arrows) and as side views in the primary channel (yellow circle, right) or into the secondary channel (green circle; left). B) Representation of the 3D-structure of PqsD showing a water-filled (red spheres) central cavity (in green the flexible regions of PqsD are shown, which are defined according to b-factors and to the differences in the three crystal structures). In the zoomed-view of the central cavity the polar network of "substrate-loop" residues His86 and Asp87 with Ser317' of the second chain is shown, which is repeated symmetrically on the opposite chain. These three residues could form a thioesterase triad as suggested by their geometric disposition and by their distances d_1 and d_2.

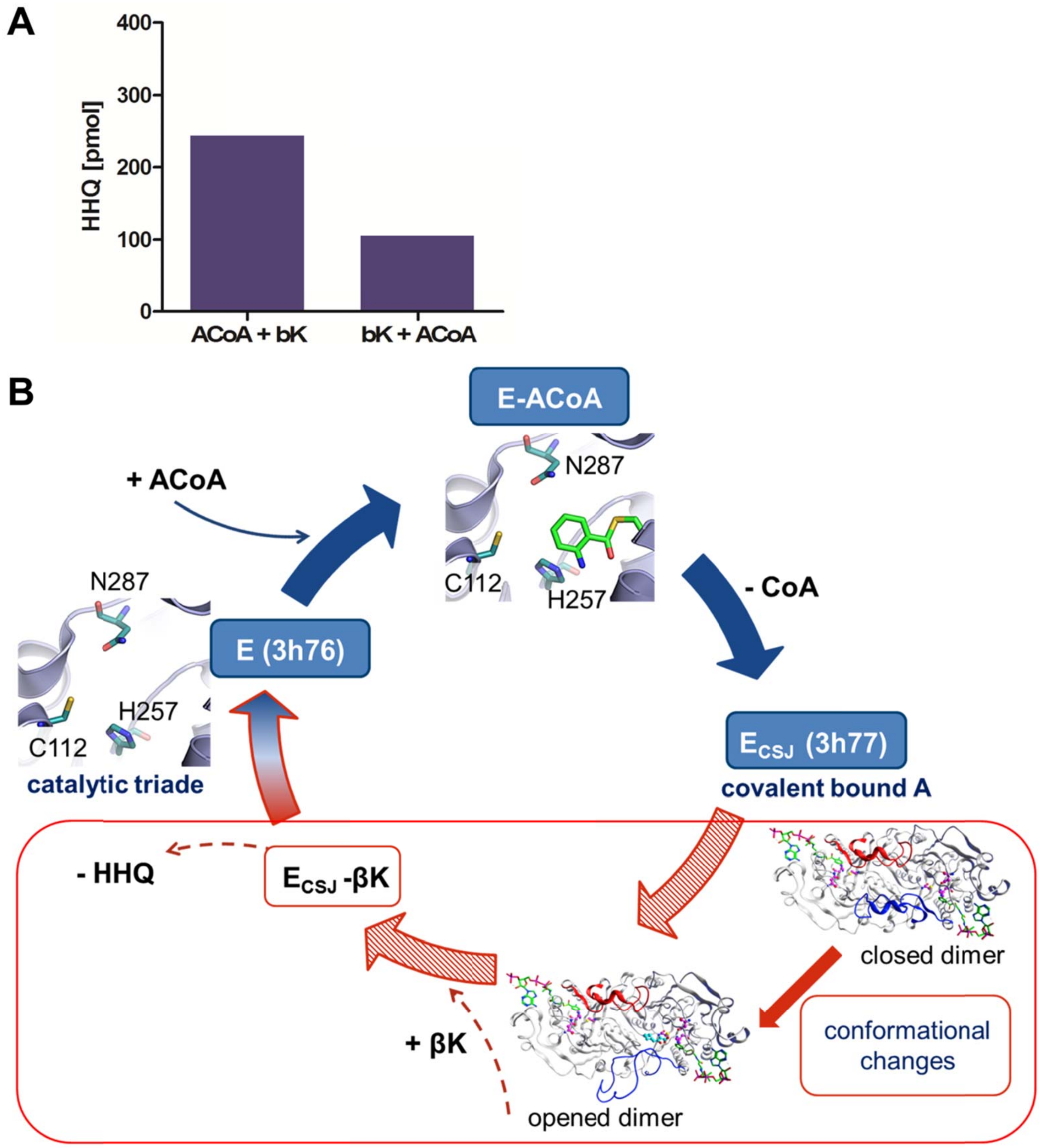

FIGURE SI2 - HHQ biosynthesis follows a ping-pong mechanism. A) Time-dependent HHQ formation as function of the substrate addition order determined by SPR experiments. About double amount of HHQ is collected when ACoA is added before βK. B) Proposed kinetic cycle: steps linked by blue arrows are supported by existing crystallographic data, steps linked by red arrows are hypothetical and are generated by computational methods (i.e. docking and modelling).

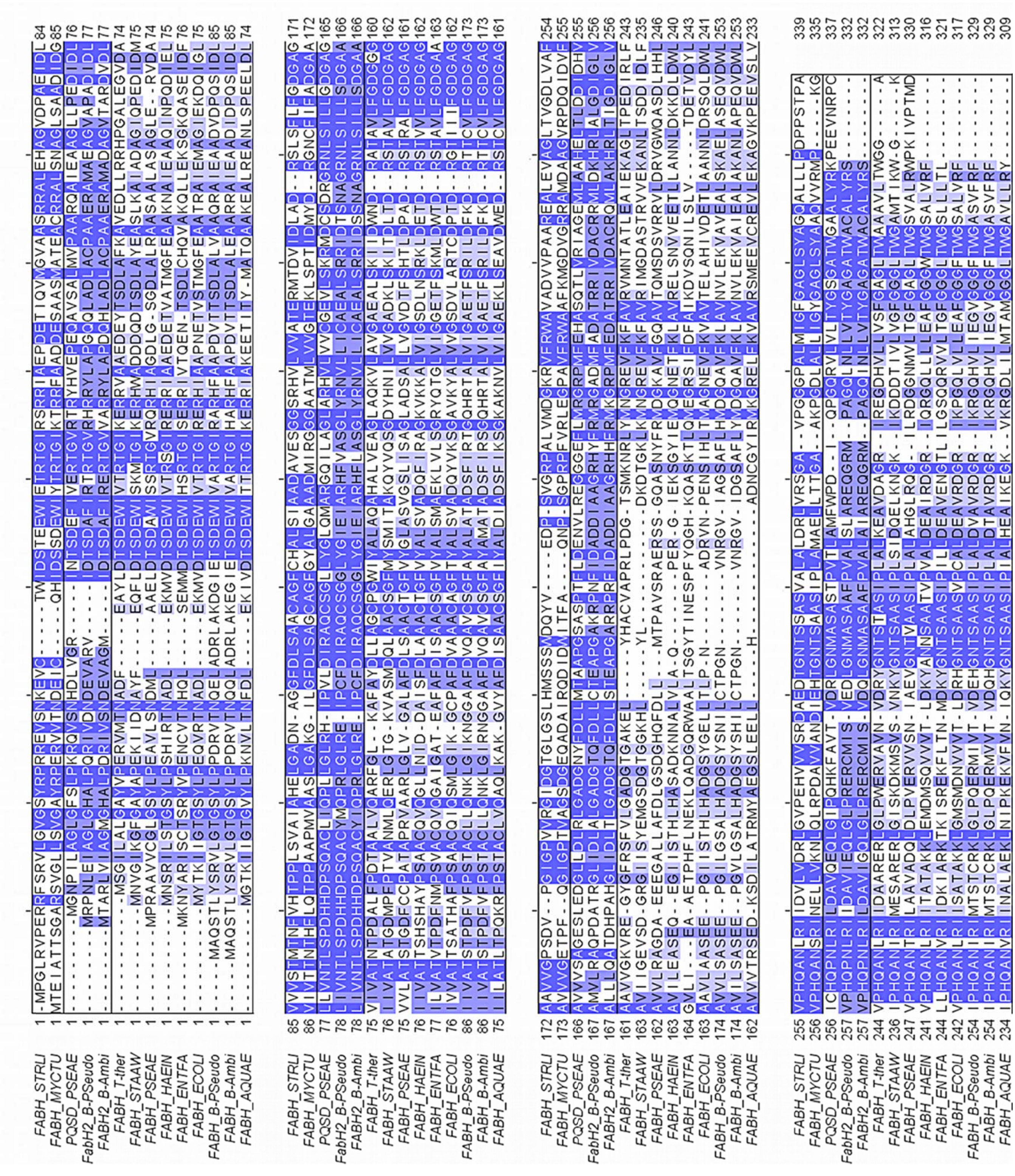

FIGURE SI3 - Multiple sequence alignment of PqsD and KAS III enzymes. Abbreviations: *PSEAE – Pseudomonas aeruginosa*, *B-Ambi – Burkholderia ambifaria*, *B-PSeudo – Burkholderia* pseudomallei, *ECOLI – Eschericchia coli*, *HAEIN – Haemophilus influenzae*, *AQUAE – Aquifex aeolicus*, *T-ther – Thermus thermophilus*, *STAAW – Staphilococcus aureus*, *ENTFA – Enterococcus faecalis*, *STRLI – Streptomyces lividans*, *MYCTU – Mycobacterium tuberculosis*.

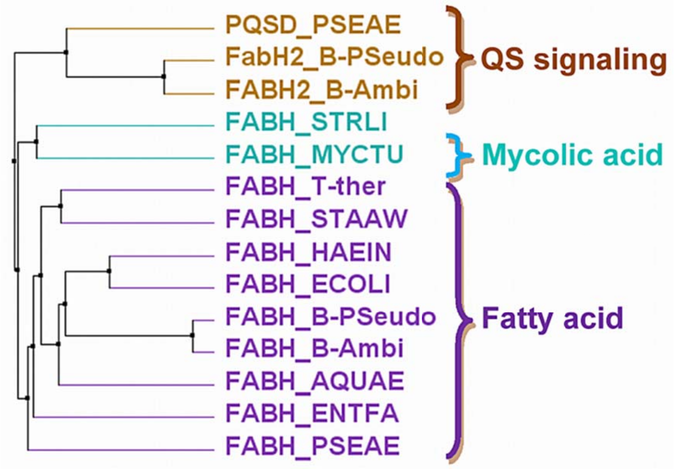

FIGURE SI4 - Average distance tree using PID (% identity) from the ProconsWS alignment. The fourteen enzymes are classified into three groups (color-coded in tan, cyan, magenta) corresponding to the peculiarities of the biosynthetic products of the strains. Abbreviations: *PSEAE – Pseudomonas aeruginosa*, *B-Ambi – Burkholderia ambifaria*, *B-PSeudo – Burkholderia* pseudomallei, *ECOLI – Eschericchia coli*, *HAEIN – Haemophilus influenzae*, *AQUAE – Aquifex aeolicus*, *T-ther – Thermus thermophilus*, *STAAW – Staphilococcus aureus*, *ENTFA – Enterococcus faecalis*, *STRLI – Streptomyces lividans*, *MYCTU – Mycobacterium tuberculosis*.

We performed a BLAST search of PqsD (UniProt-ID P20582) against the Uniprot database and the PDB database (excluding hits within sequence identity (s.i.) of >95%). The PDB-search identified FabHs of Gram-positive and Gram-negative bacteria (s.i. ~29-40%) as the closest enzymes; for the Uniprot-search FabH2 of *B. pseudomallei* and *B. ambifaria* (~55% s.i.) were found. A multiple sequence alignment of PqsD with the top ten PDB-search hits, FabH2 of *B. ambifaria* and *B. pseudomallei,* and FabH of *P. aeruginosa* (Fig. SI3) was performed using Probcons with Jalview. The average distance tree divided these enzymes in three groups, each with distinct final products and biosynthesis pathway (Fig. SI4).

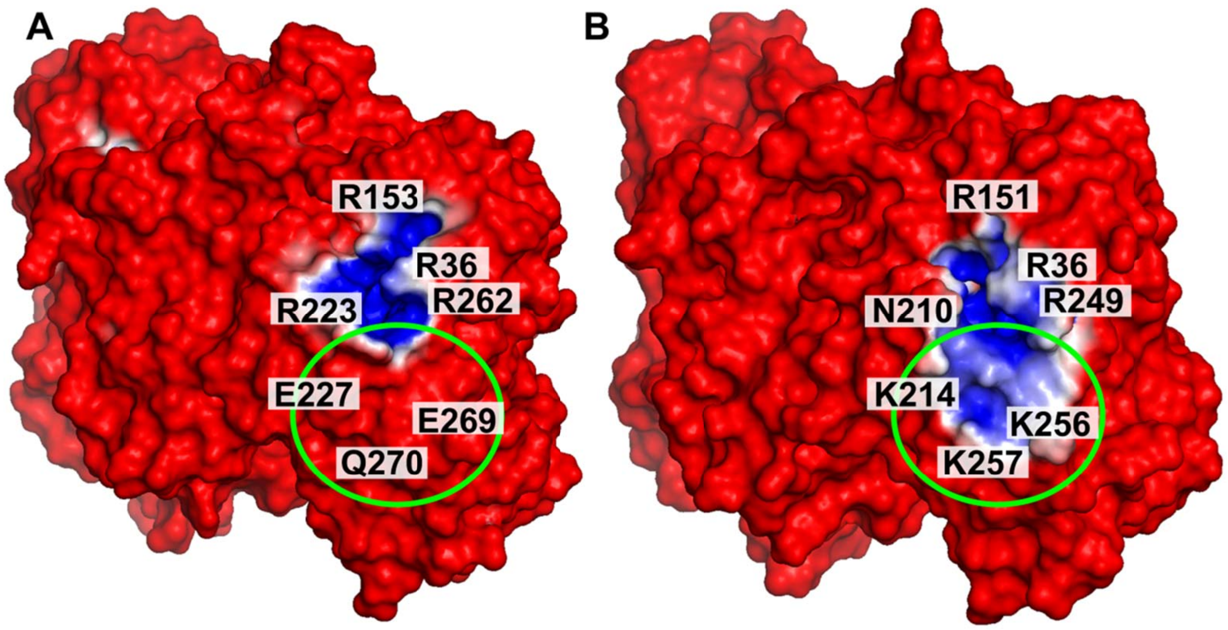

FIGURE SI5 - ±2 kT/e electrostatic potential of PqsD (A) and E. coli FabH (B) plotted on the solvent-accessible surface, computed with APBS [5] and visualized with PyMOL. The green circle evidences the area in which the two enzymes mainly differ, in particular, due to the absence of cationic residues in PqsD replaced by either acidic (E227 and E269) or neutral (Q270) amino acids. The modified electrostatic potential suggests that ACP will bind to PqsD covering a different region.

A

HingeMaster prediction for apoform PqsD crystal structure PDB-id 3H76.

HingeMaster (red trace) combines the output of all predictors and should be used as the default predictor for most purposes. Higher values are more likely hinge points.
hNMA (dotted magenta trace) is the squared-normalized first normal mode displacement according to GNM. Values near zero of this quantity are correlated with hinge location. FO1 (dotted black trace) gives the sum of folding free energies of two fragments generated by cutting the backbone at residue i. Local minima correspond to hinges. HingeSeq (dash-dotted cyan trace) is a weak predictor based on sequence features. Lower values of this quantity reflect more likely hinge locations.

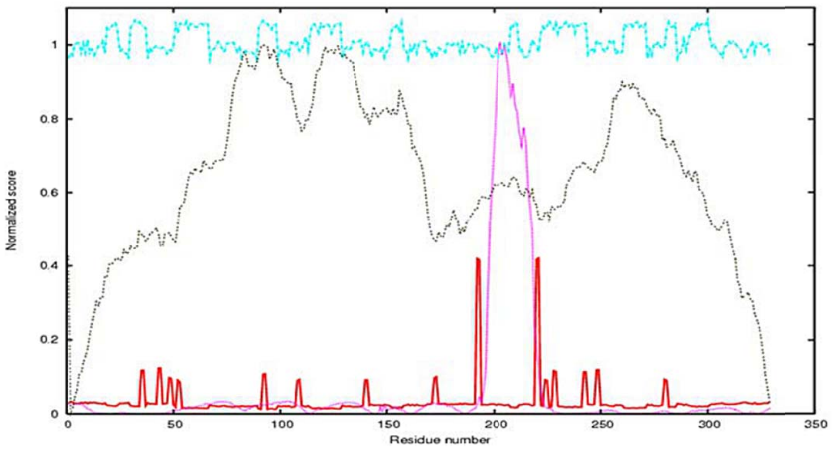

B

Two-cut FlexOracle free energies of folding.

Graph shows sum of free energies of folding using the FoldX force field, for two fragments generated by cutting at residues i (abscissa) and j (ordinate). Free energies for all possible values of i,j (excluding residues near the termini) are plotted. Local minima in this quantity, after filtering out uninteresting regions of i,j space, correspond to hinge locations. The most likely hinge is reported as FO in the "Results for discrete predictors" graph.

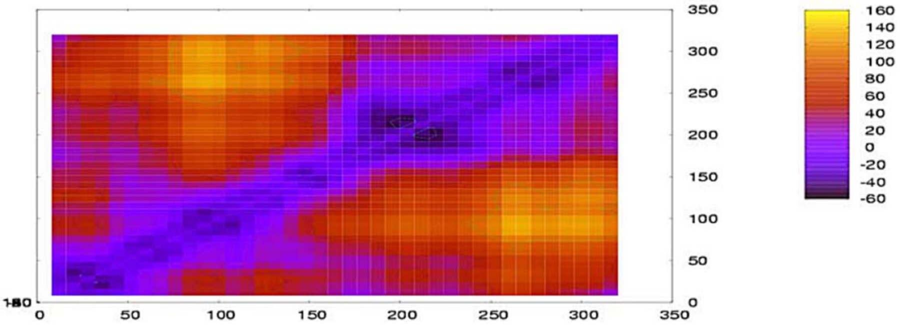

C

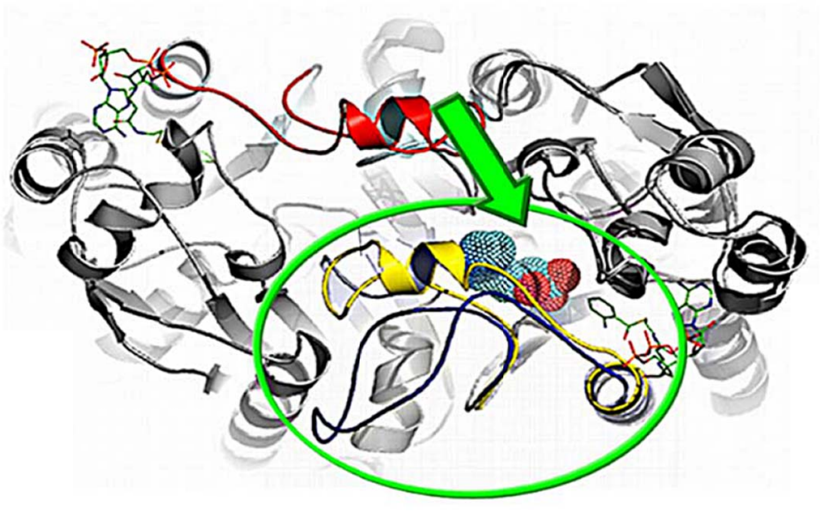

FIGURE SI6 - Predicted hinge regions in PqsD. A) HingeMaster prediction; B) Two-cut FlexOracle free energies of folding; C) Top view of the dimer interface of closed (yellow) and open conformer (blue; modelled according to hinge-predictions) of PqsD. The hairpin loop of the second, closed, chain is coloured in red. βK is rendered as red and cyan dots in its modelled pose in the secondary channel (green arrow), ACoA molecules in the primary funnels are shown as sticks.

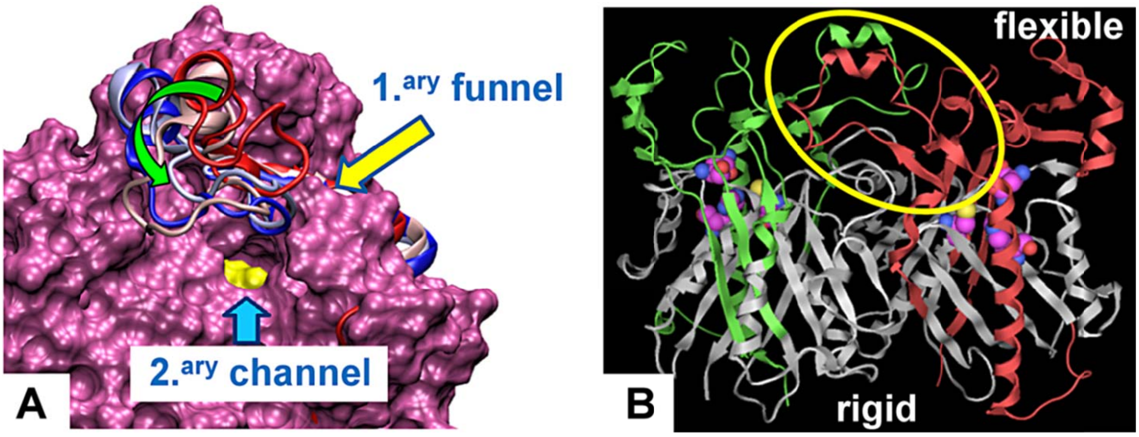

FIGURE SI7 - PqsD flexibility in the single monomer MD simulations. A) Side-view of four superimposed snapshots of the monomer MD **A** with the **hL** (cartoons, color-coded red to blue: 0, 10, 20, 30 ns) collapsed (green arrow). B) Flexible regions of PqsD evidenced in red and green with **hL** (residues 185-220) as yellow circle.

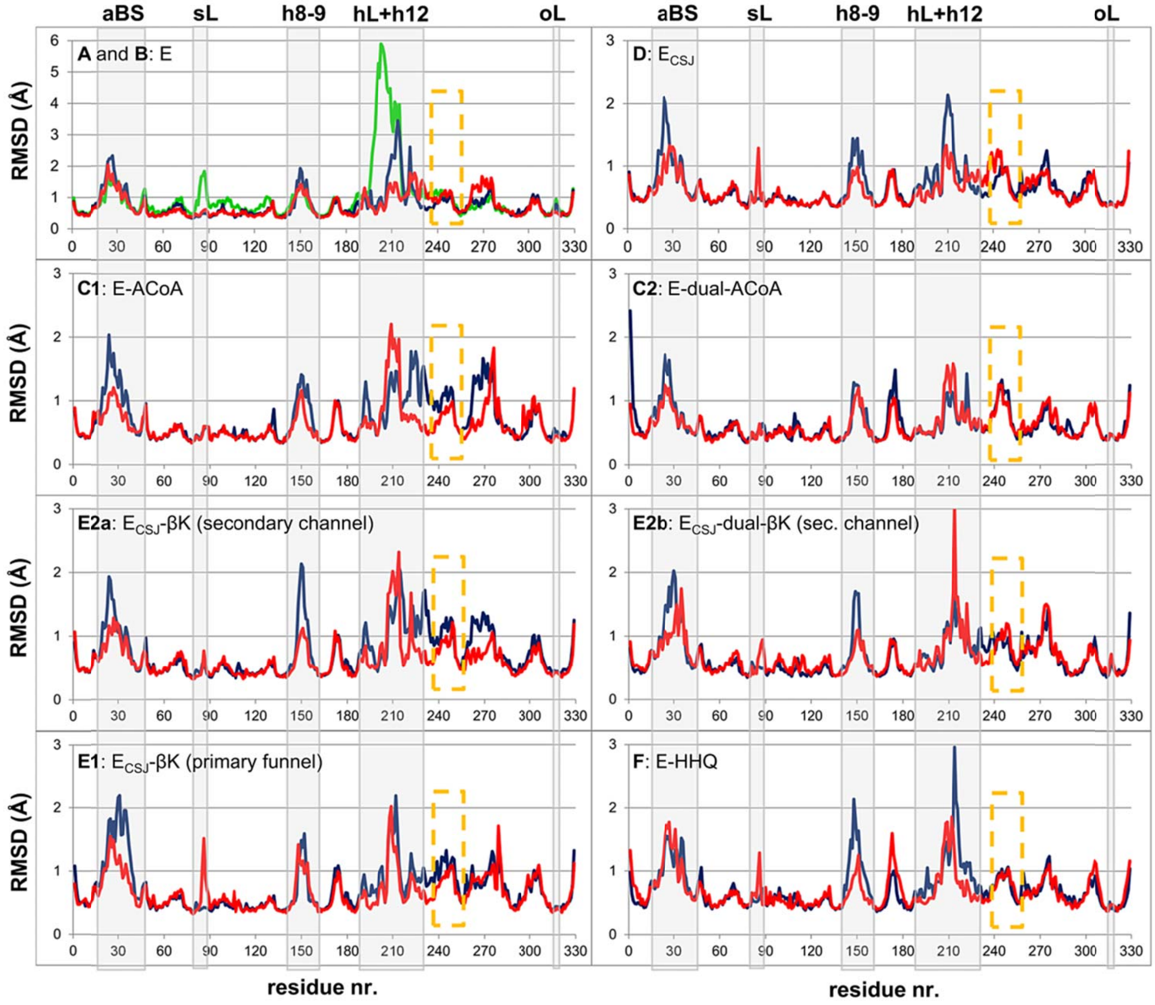

FIGURE SI8 - Residue-dependent RMS fluctuations (RMSF) for the MD simulations **A**-**F**. For each plot chain A of PqsD is coloured in blue (the monomeric MD **A** is shown in green) and chain B in red. Putative catalysis-involved domains identified by comparative analysis with homologue KAS-III enzymes are labelled with their abbreviations (dotted orange box indicates helix H14): adenosine binding site -**aBS**, palindromic "substrate-loop" - **sL**, helix H8-H9 loop - **h8-9**, hairpin-loop - **hL**, helix H12 - **h12**, "oxyanion-loop" – **oL**. The largest RMSF amplitudes are all localized within these domains.

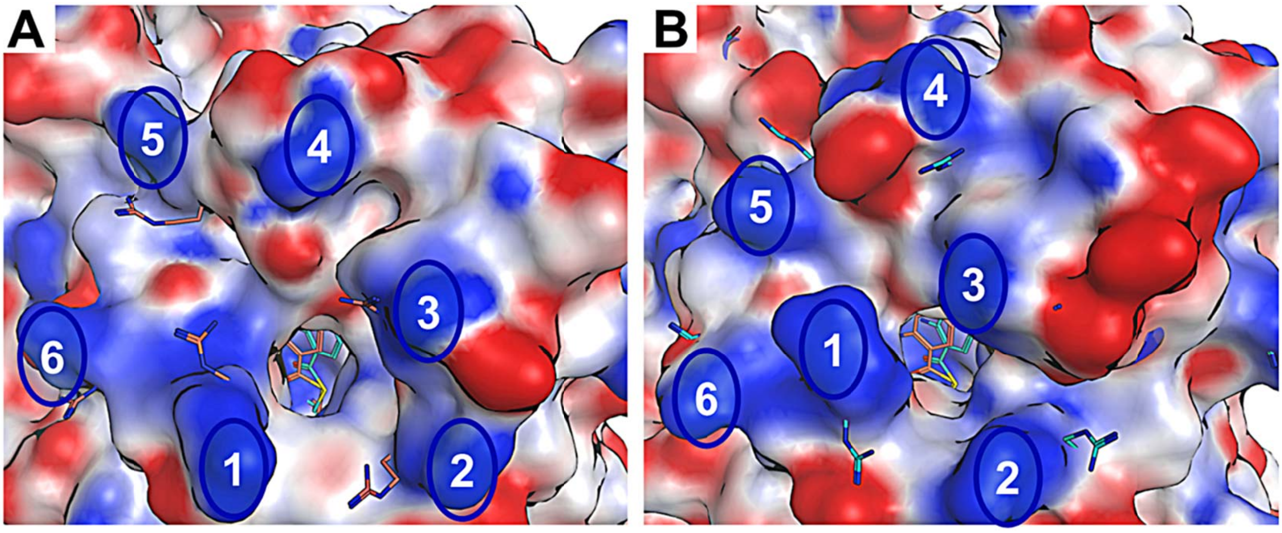

FIGURE SI9 - Conformational changes of the cationic belt. Side-view of the molecular surface of the cationic belt evidencing the different positions of arginines 223 (1), 262 (2), 36 (3), 153 (4), 151 (5) and 221 (6) after 0 **ns** (A) and 30 ns (B) of the dual-βK MD simulation **E2b**.

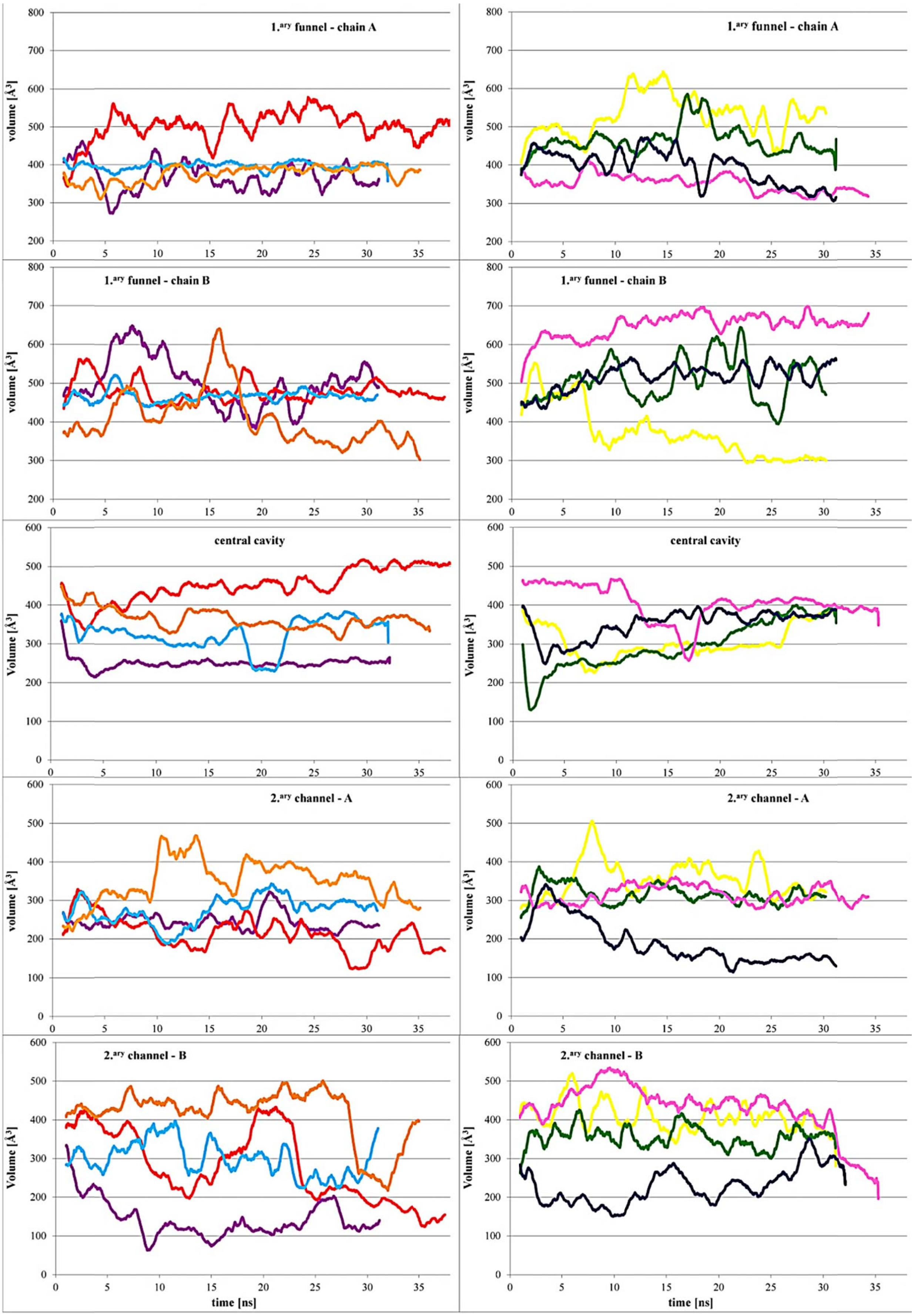

FIGURE SI10 - Time-dependent volume variations of primary funnel and secondary channel of chain A and B and of the central cavity of MD simulations **B-F** determined with fpocket2 [4]. On the left side we compared the MD simulations **B** (apoform; purple), **C1** (single-ACoA; red), **C2** (dual-ACoA; marine blue), and **D** (CSJ-PqsD; orange), whereas on the right side the MDs **E1** (βK in primary funnel; pink), **E2a** (single_βK in secondary funnel; yellow), **E2b** (dual_ βK in secondary funnel; dark green), and **F** (HHQ; dark blue).

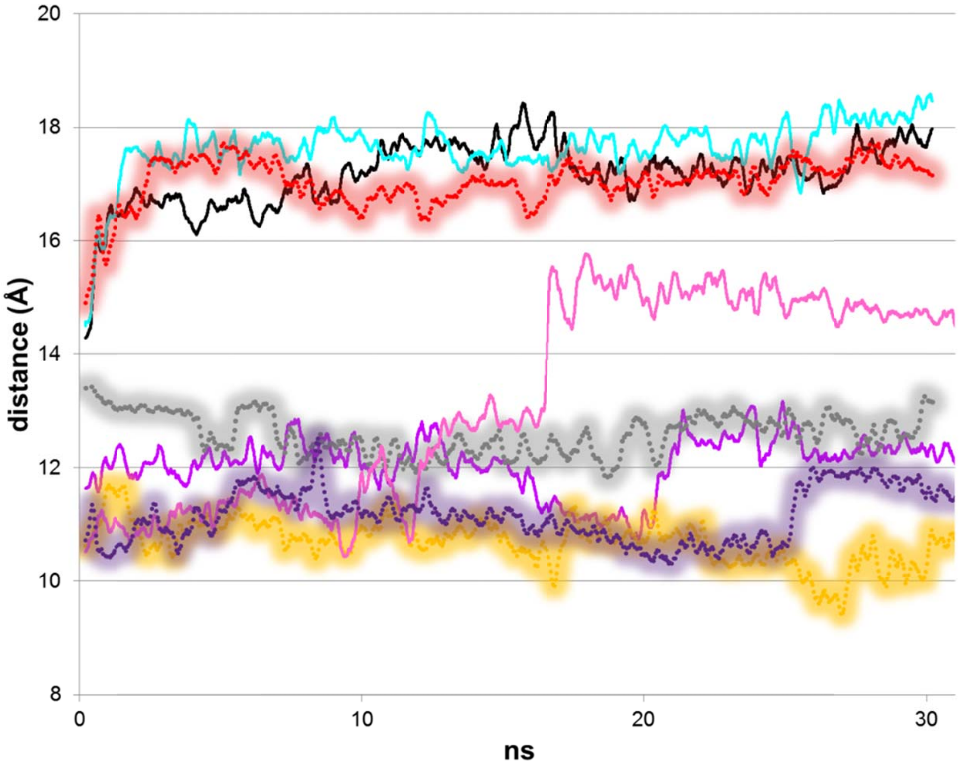

FIGURE SI11 - Time-dependent distance variation between Phe218 and Cys112. The distance variation over the time between the centre of mass of the phenyl ring and the C_{α} atom of Cys112 for the MD simulations **C2** (dual_ACoA; chain A – magenta, chain B – light orange), **E2a** (βK in secondary channel of chain A; chain A – black, chain B – grey), **E2b** (βK in secondary channel; chain A – cyan, chain B – red), and **F** (HHQ in chain B; chain A – pink, chain B – dark purple) are shown as running averages with a period of 200. Chains A are shown as solid lines, whereas chains B as round dots with transparent glow.

A

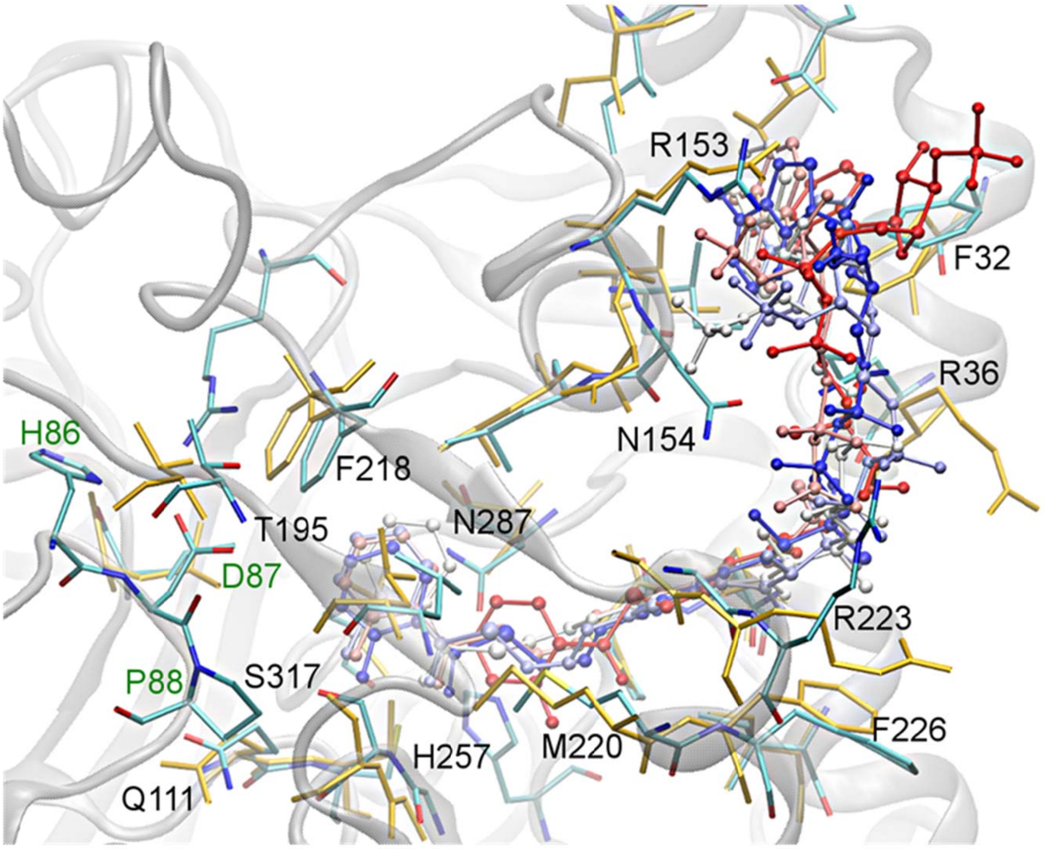

B

	mono_ACoA				bis_ACoA			
	chain A		chain B		chain A		chain B	
	**initial**	**final**	**initial**	**final**	**initial**	**final**	**initial**	**final**
CSJ112_S-N287	5.3	5.9	5.8	**4.8**	6.3	8.0	8.2	7.7
CSJ112_S-H257	4.1	8.8	4.1	**3.4**	4.4	**3.6**	4.6	4.7
H257-N287	6.0	9.3	5.7	**4.3**	6.6	6.0	6.3	5.6

C

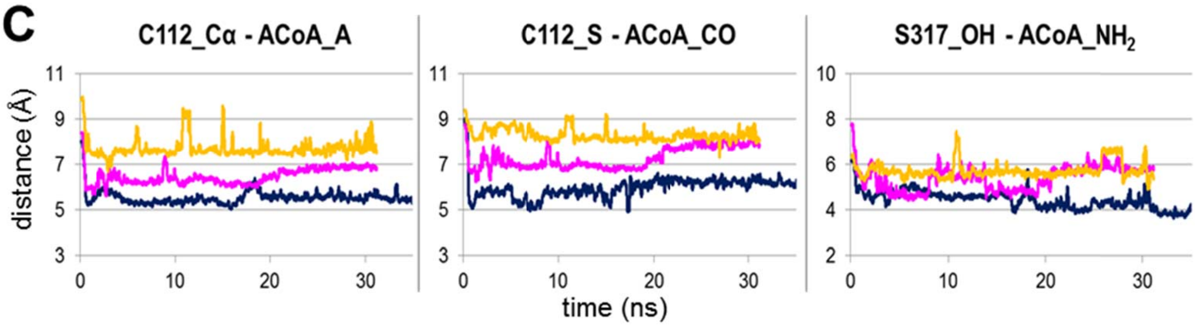

FIGURE SI12 - Binding mode of ACoA in MD simulations **C1** and **C2**. A) Progression of ACoA in the single-ACoA MD simulation **C1** (ACoA is shown as ball and sticks color-coded as follows: 0 - red, 10- rosa, 20 - white, 30 ns – light blue, 34 ns - blue). Surrounding residues at 0 (yellow) and 34 ns (cyan) are shown as sticks. B) Initial and final distances (in Å) between the three catalytic residues are listed; the distances were measured between the sulphur atom of Cys112 (C112_S) and the centres of masses of ND1-CE1-NE2 of His257 and of the amide group of Asn287, respectively. C) Distances between the Cα of Cys112 and the centre of mass of the anthranilic moiety (ACoA_A), between C112_S and the centre of mass of the thioester carbonyl (ACoA_CO), and between OH of Ser317 and the amide of ACoA (ACoA_NH_2) are compared for the MD simulations **C1** (dark blue) and **C2** (ACoA; chain A – magenta, chain B – dark yellow).

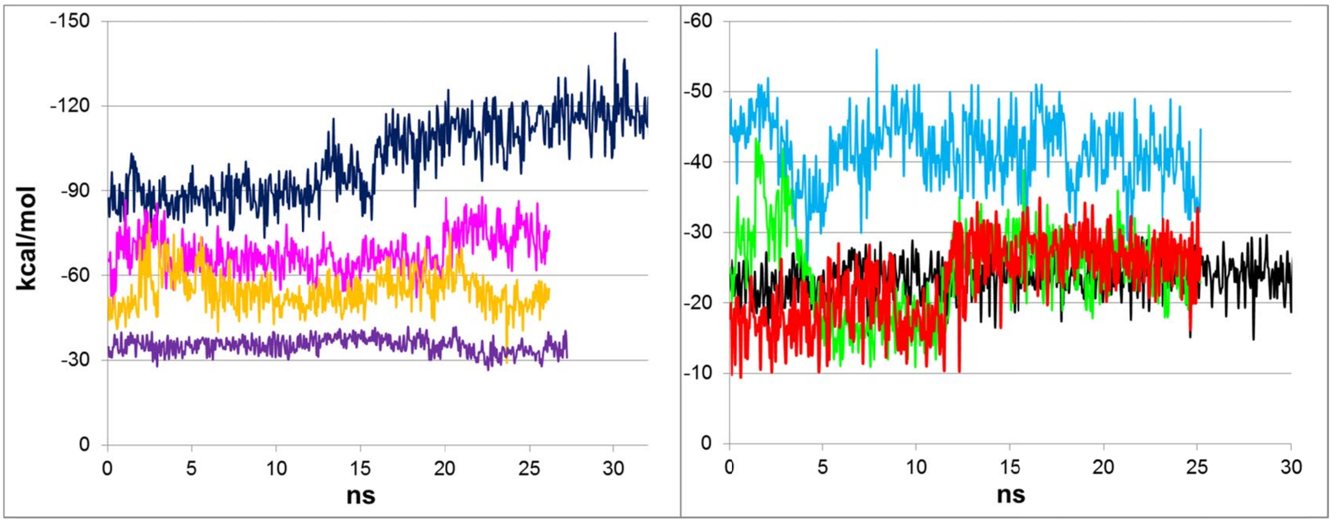

FIGURE SI13 - Time-dependent variation of the estimated binding energy ΔG_{bind} using MM-GBSA methods. On the left side ΔG_{bind} (kcal/mol; enthalpy only) is plotted for the MD simulations **C1** (ACoA; dark blue), **C2** (ACoA; chain A – magenta, chain B – dark yellow), and **F** (HHQ; dark purple). On the right side ΔG_{bind} of **E1** (βK in primary funnel; black), **E2a** (βK in secondary channel; green) and **E2b (**βK in secondary channel; chain A - cyan, chain B - red) are shown. For all MD simulations the first 5 ns of the production run were discarded for the ΔG_{bind} determination.

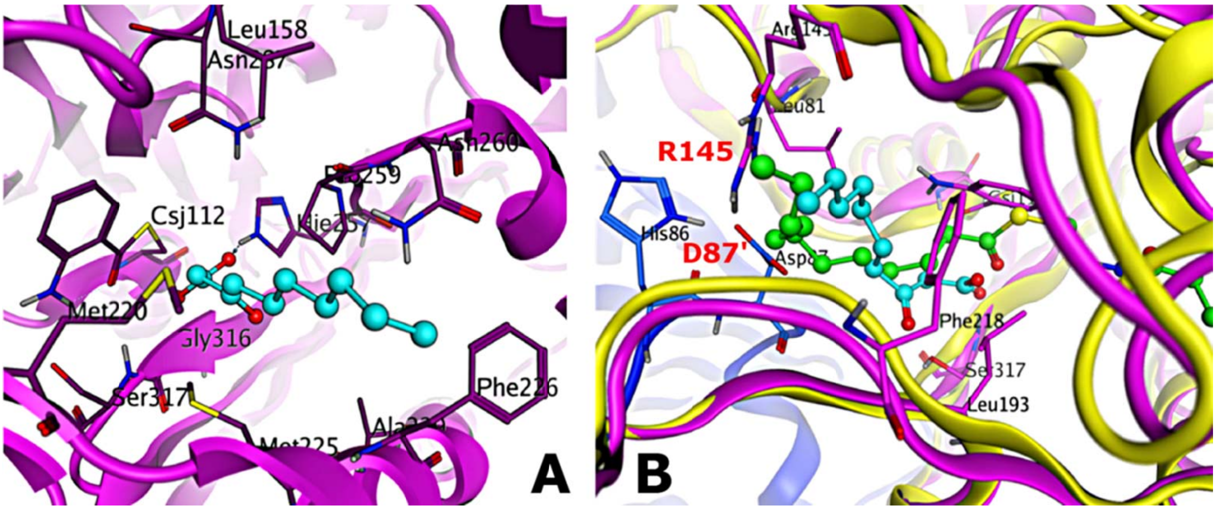

FIGURE SI14 - Where does βK bind in PqsD? **A**) Docking pose of βK into the primary channel of chain B. **B**) Modelled βK (cyan) pose in the secondary channel of chain A of PqsD (magenta) after superimposition with *M. tuberculosis* FabH (yellow) and merging of dodecyl-CoA (green). His86-Asp87-Ser317' are rendered as sticks, while βK and dodecyl-CoA as ball-and-sticks. The ion-pair D87'-R145, which forms one wall of the secondary channel, is evidenced by red labels.

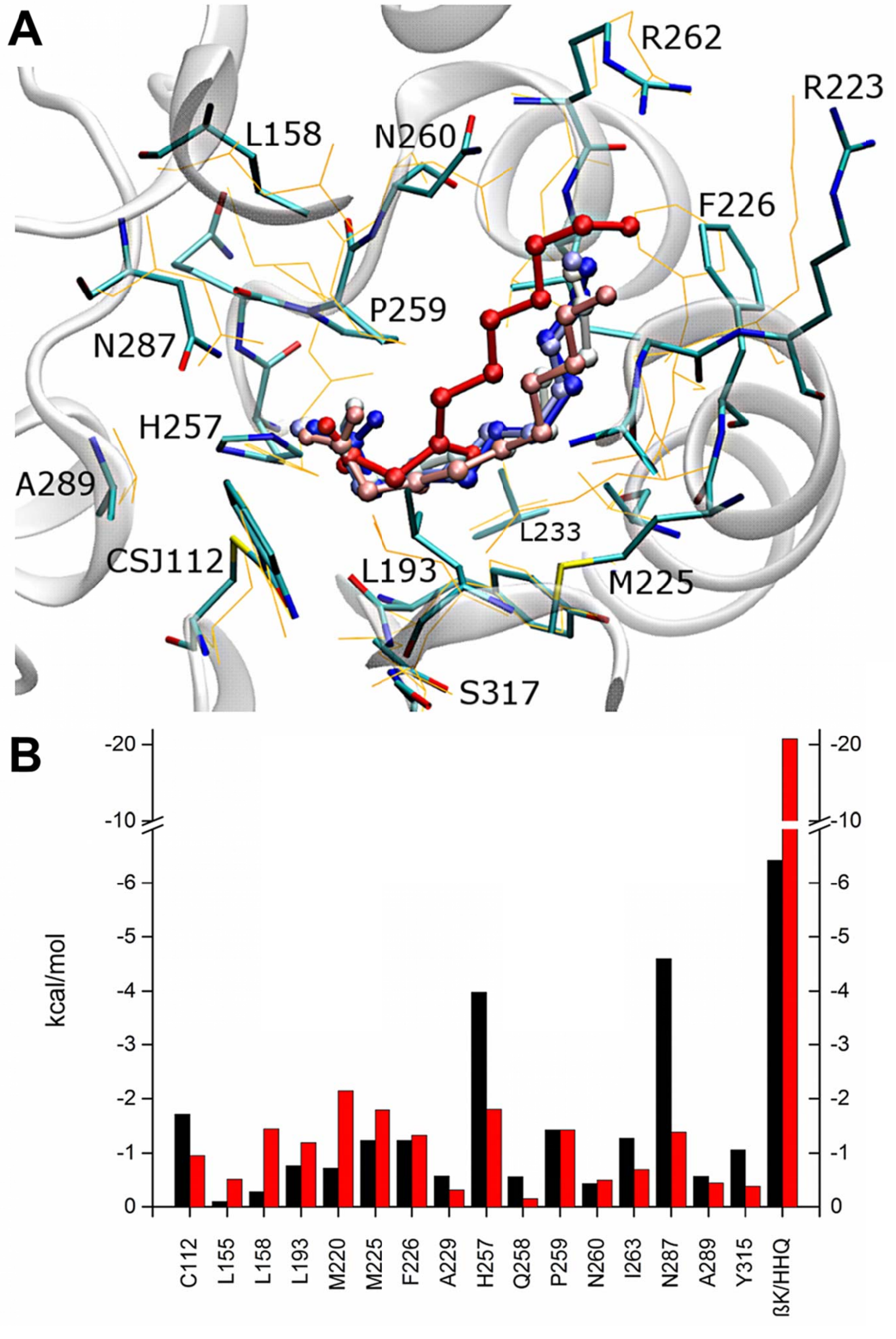

FIGURE SI15 - Binding mode of βK in the primary funnel of chain B of the MD simulation **E1**. A) Snapshots at 0 (red), 10 (pink), 20 (white), 30 (light blue) and 34 (blue) ns of the single-βK MD simulations **E1** with βK in the primary funnel of chain B of the CSJ-PqsD complex. Surrounding residues are shown as cyan sticks (34 ns) and orange lines (0 ns). B) Decomposed energy contributions per residue (at least for one MD >0.5 kcal/mol) determined by MM-GBSA methods for the MD simulations **E1a** (black) and **F** (HHQ; red).

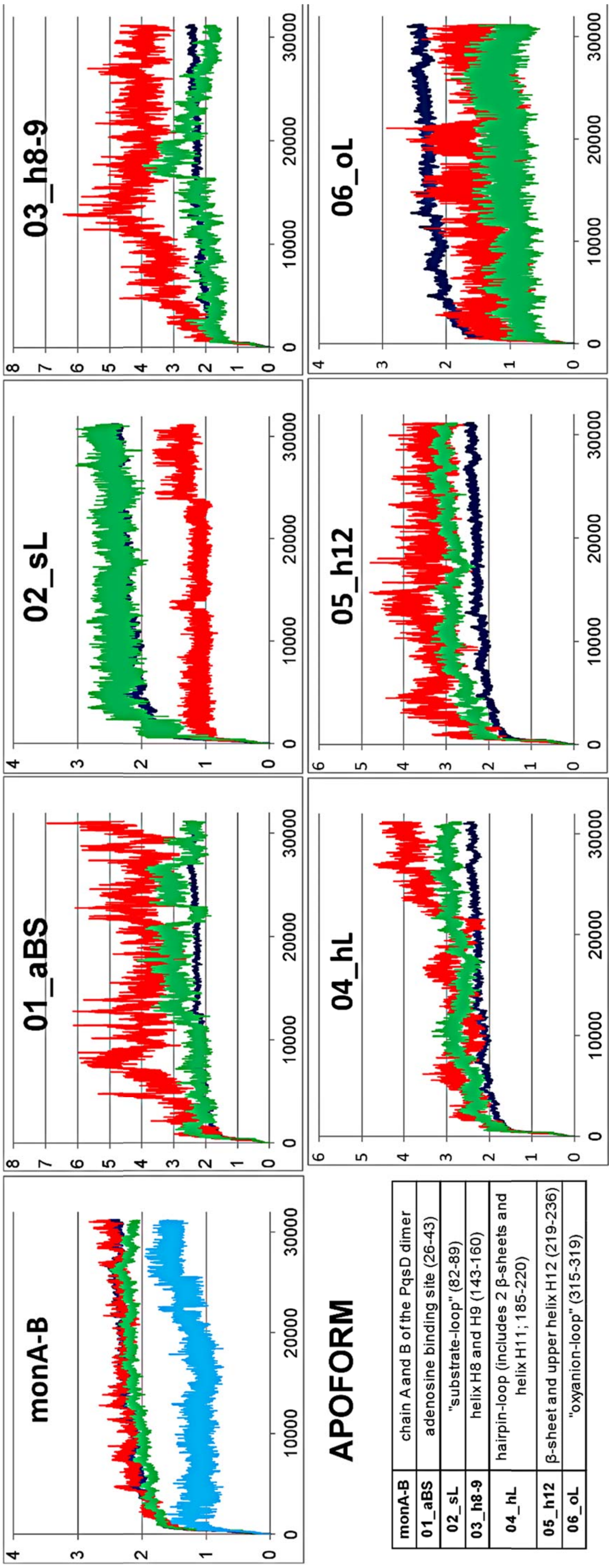

FIGURE SI16 - Trajectory analysis of the apoform MD simulation **B** in terms of time-dependent all-heavy atom RMSD fluctuation for PqsD dimer (dark blue), chain A (red) and B (green) reported for all 327 residues of each chain (**monA-B**) and focused on the putative catalysis-involved domains identified by comparative analysis with homologue KAS-III enzymes: adenosine binding site (**01_aBS**), palindromic "substrate-loop" (**02_sL**), helix H8-H9 loop (**03_h8-9**), hairpin-loop (**04_hL**), helix H12 (**h12**) and "oxyanion-loop" (**06_oL**).

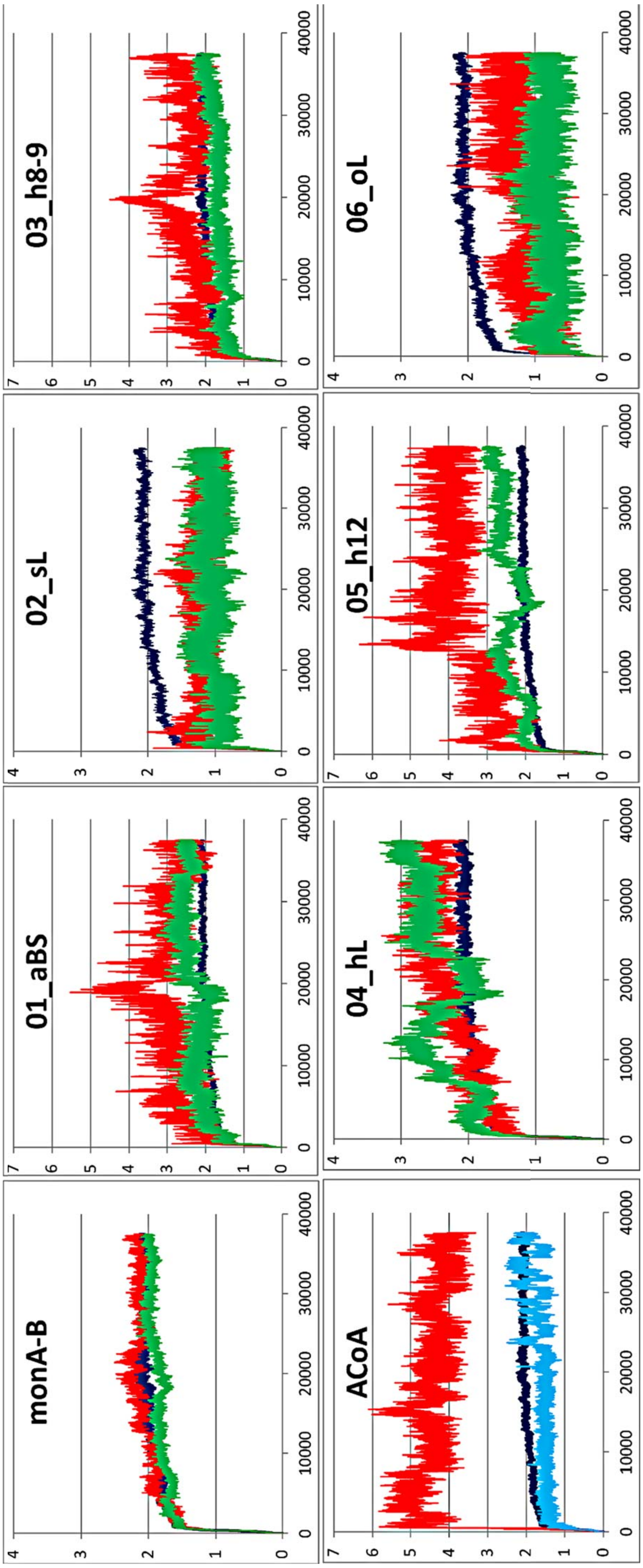

FIGURE SI17 - Trajectory analysis of the single-ACoA MD simulation **C1** in terms of time-dependent all-heavy atom RMSD fluctuation for PqsD dimer (dark blue), chain A (red) and B (green) reported for all 327 residues of each chain (**monA-B**), ACoA in chain B (**ACoA**; catalytic triad RMSD shown in light blue) and focused on the putative catalysis-involved domains identified by comparative analysis with homologue KAS-III enzymes: adenosine binding site (**01_aBS**), palindromic “substrate-loop” (**02_sL**), helix H8-H9 loop (**03_h8-9**), hairpin-loop (**04_hL**), helix H12 (**h12**) and “oxyanion-loop” (**06_oL**).

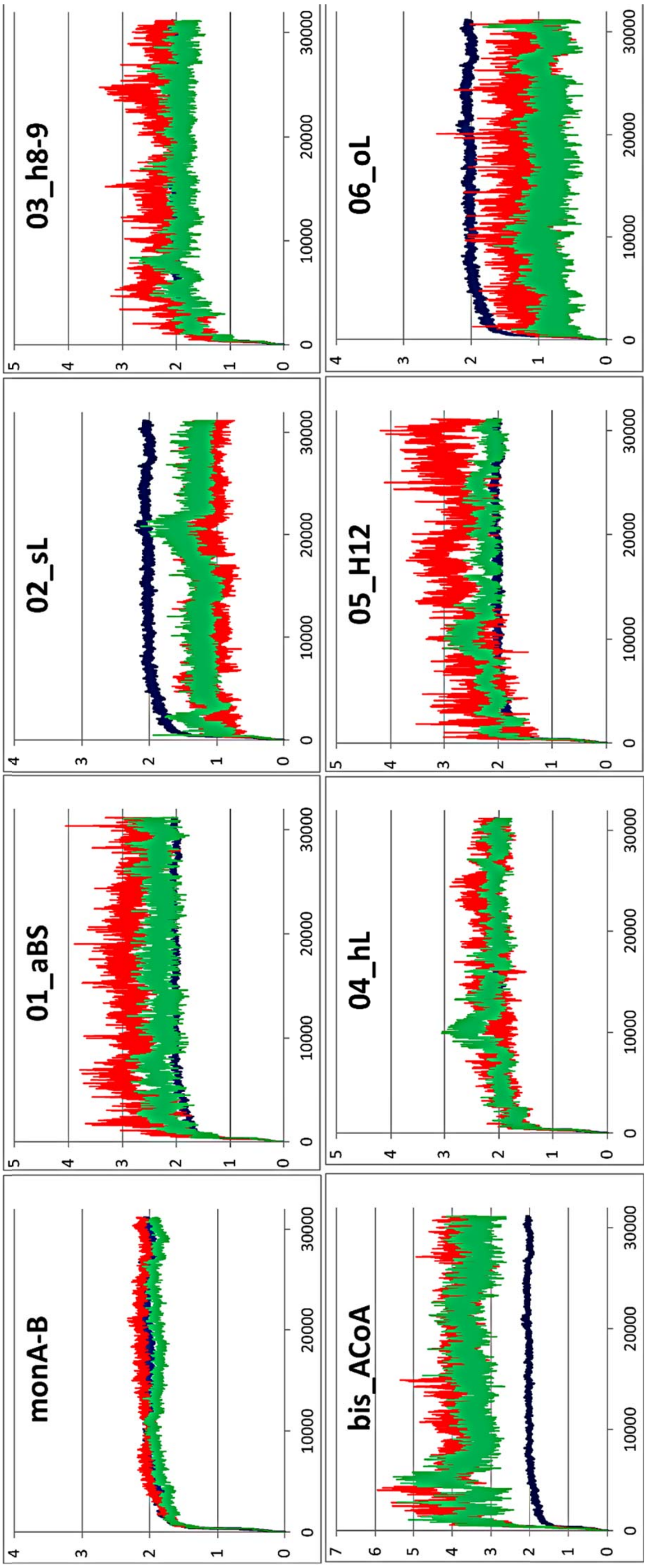

FIGURE SI18 - Trajectory analysis of the dual-ACoA MD simulation **C2** in terms of time-dependent all-heavy atom RMSD fluctuation for PqsD dimer (dark blue), chain A (red) and B (green) reported for all 327 residues of each chain (**monA-B**), both ACoA molecules (**bis-ACoA**) and focused on the putative catalysis-involved domains identified by comparative analysis with homologue KAS-III enzymes: adenosine binding site (**01_aBS**), palindromic "substrate-loop" (**02_sL**), helix H8-H9 loop (**03_h8-9**), hairpin-loop (**04_hL**), helix H12 (**h12**) and "oxyanion-loop" (**06_oL**).

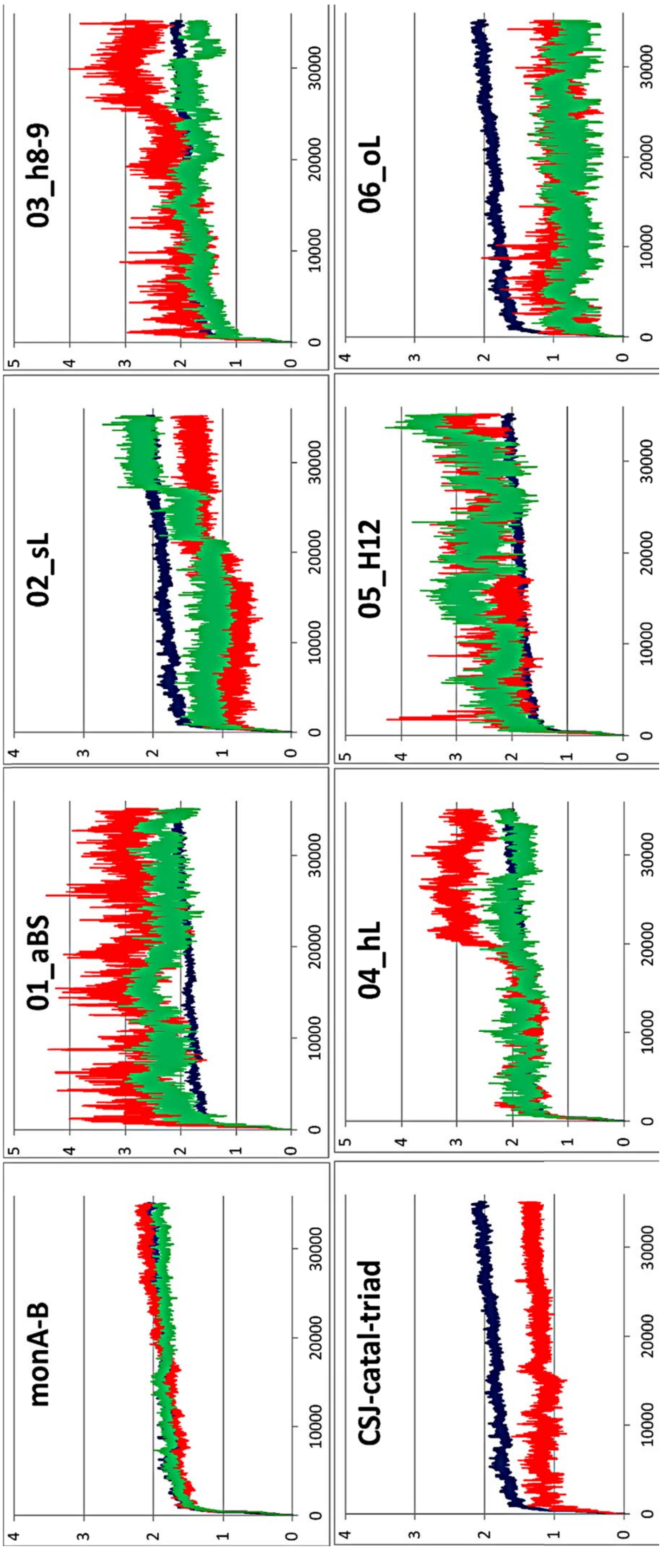

FIGURE SI19 - Trajectory analysis of the anthranilate-ligated (CSJ) MD simulation **D** in terms of time-dependent all-heavy atom RMSD fluctuation for PqsD dimer (dark blue), chain A (red) and B (green) reported for all 327 residues of each chain (**monA-B**), catalytic triads including CSJ (**CSJ-catal-triad**, red) and focused on the putative catalysis-involved domains identified by comparative analysis with homologue KAS-III enzymes: adenosine binding site (**01_aBS**), palindromic “substrate-loop” (**02_sL**), helix H8-H9 loop (**03_h8-9**), hairpin-loop (**04_hL**), helix H12 (**h12**) and “oxyanion-loop” (**06_oL**).

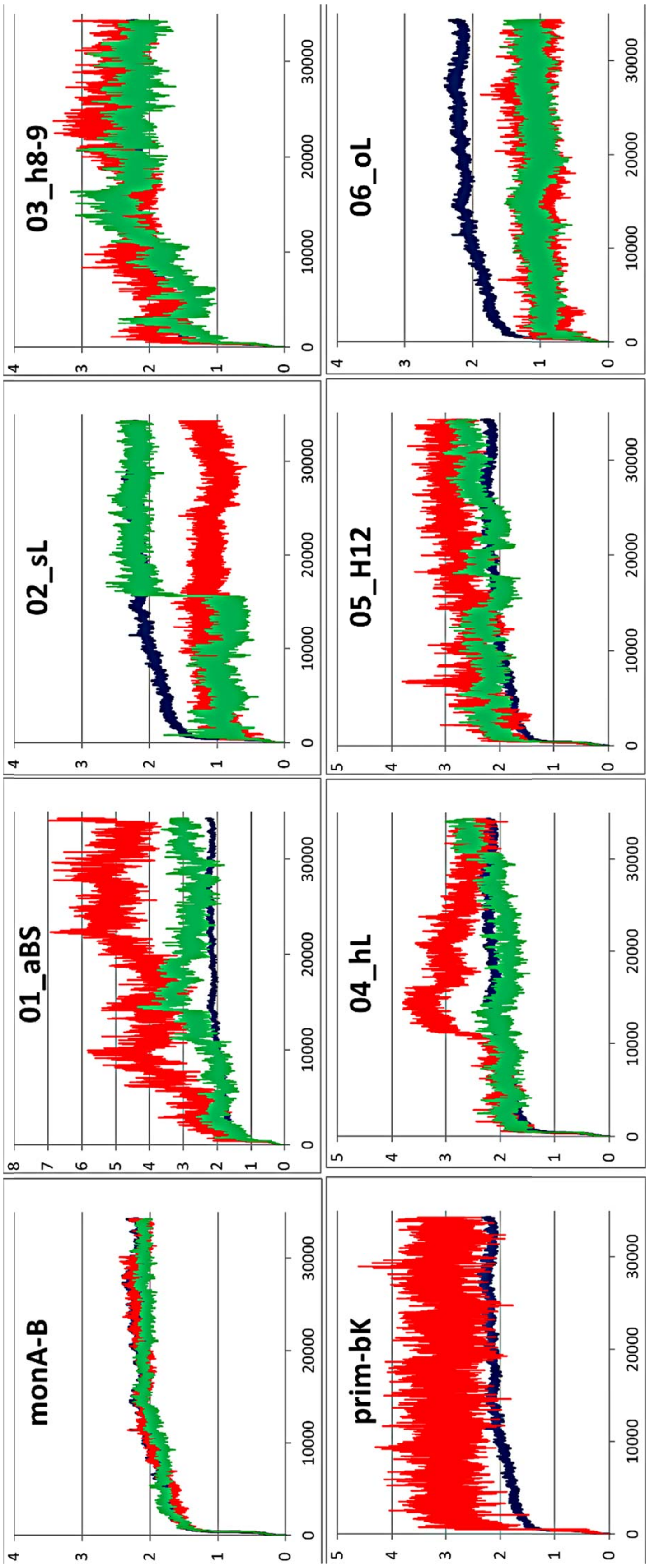

FIGURE SI20 - Trajectory analysis of the mono-βK MD simulation **E1** (βK in primary funnel of chain B) in terms of time-dependent all-heavy atom RMSD fluctuation for PqsD dimer (dark blue), chain A (red) and B (green) reported for all 327 residues of each chain (**monA-B**), βK in chain B (**prim_bK**) and focused on the putative catalysis-involved domains identified by comparative analysis with homologue KAS-III enzymes: adenosine binding site (**01_aBS**), palindromic "substrate-loop" (**02_sL**), helix H8-H9 loop (**03_h8-9**), hairpin-loop (**04_hL**), helix H12 (**h12**) and "oxyanion-loop" (**06_oL**).

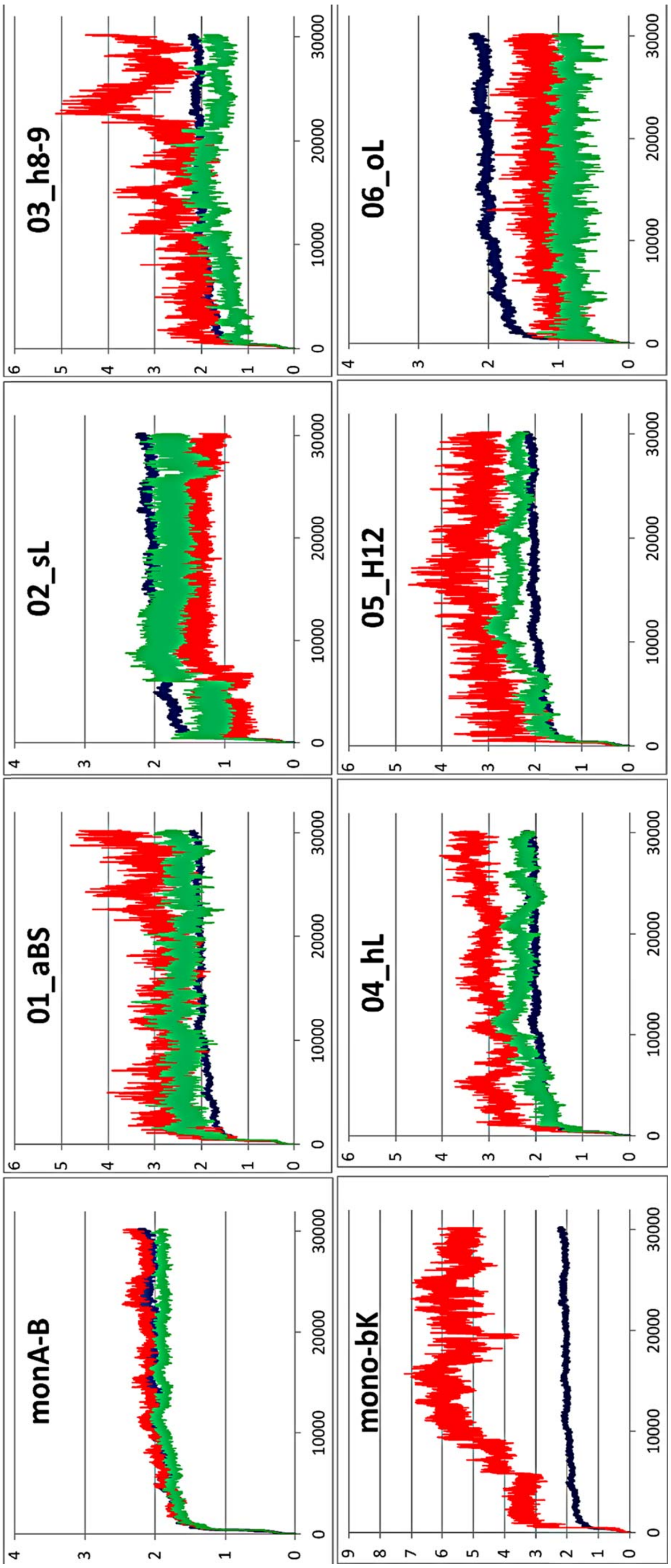

FIGURE SI21 - Trajectory analysis of the mono-βK MD simulation **E2a** (βK in secondary channel of chain A) in terms of time-dependent all-heavy atom RMSD fluctuation for PqsD dimer (dark blue), chain A (red) and B (green) reported for all 327 residues of each chain (**monA-B**), βK in chain A (red; **prim_bK**) and focused on the putative catalysis-involved domains identified by comparative analysis with homologue KAS-III enzymes: adenosine binding site (**01_aBS**), palindromic "substrate-loop" (**02_sL**), helix H8-H9 loop (**03_h8-9**), hairpin-loop (**04_hL**), helix H12 (**h12**) and "oxyanion-loop" (**06_oL**).

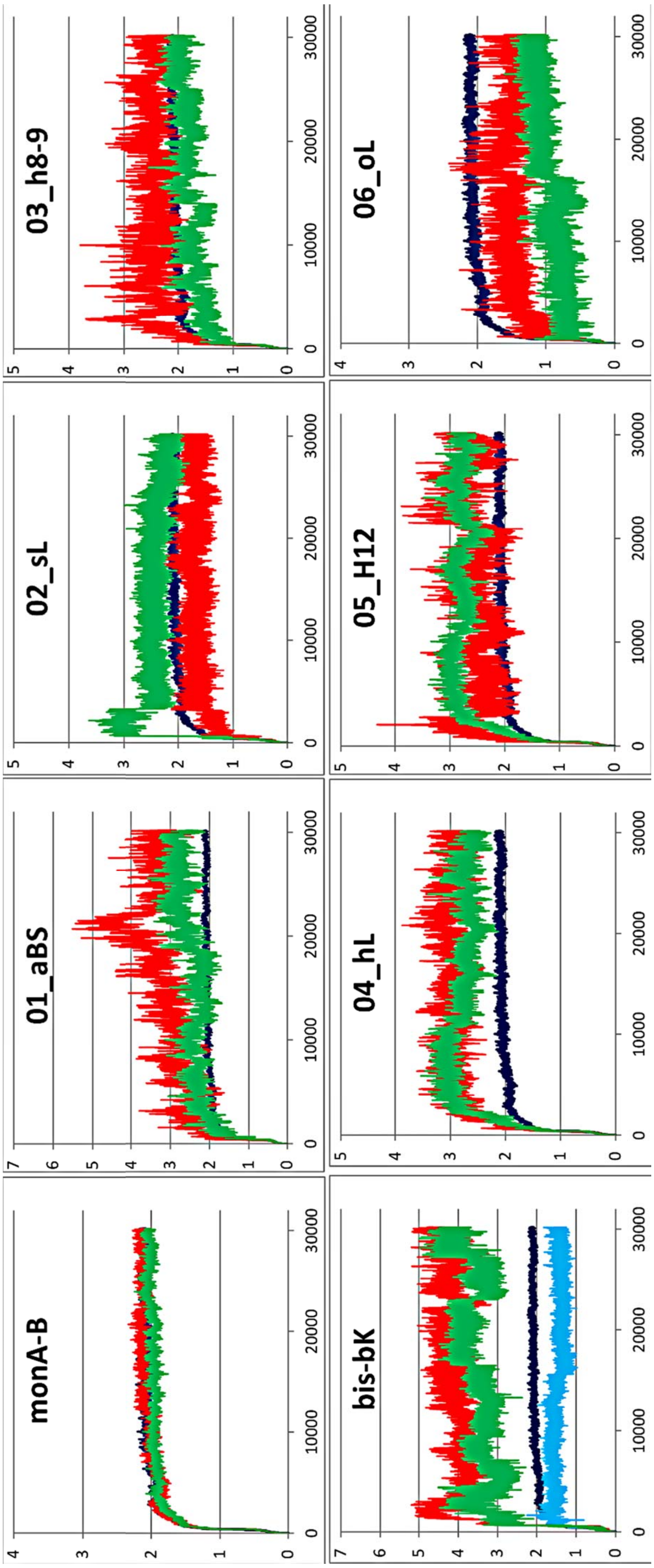

FIGURE SI22 - Trajectory analysis of the dual-βK MD simulation **E2b** (βK in secondary channel of both chains) in terms of time-dependent all-heavy atom RMSD fluctuation for PqsD dimer (dark blue), chain A (red) and B (green) reported for all 327 residues of each chain (**monA-B**), both βK molecules (**bis-bK**; red – chain A, green – chain B; catalytic triad RMSD of chain A and B shown in light blue) and focused on the putative catalysis-involved domains identified by comparative analysis with homologue KAS-III enzymes: adenosine binding site (**01_aBS**), palindromic "substrate-loop" (**02_sL**), helix H8-H9 loop (**03_h8-9**), hairpin-loop (**04_hL**), helix H12 (**h12**) and "oxyanion-loop" (**06_oL**).

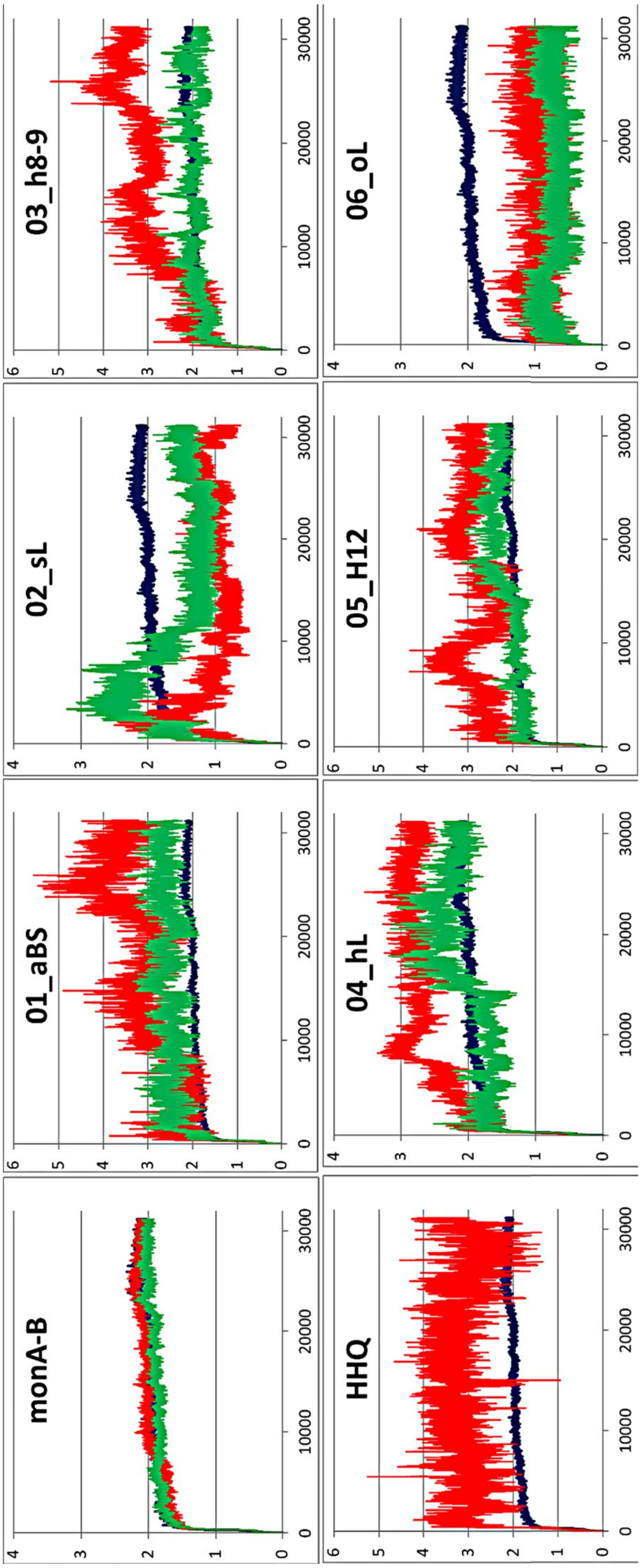

FIGURE SI23 - Trajectory analysis of the mono-HHQ MD simulation **F** (HHQ in primary funnel of chain B) in terms of time-dependent all-heavy atom RMSD fluctuation for PqsD dimer (dark blue), chain A (red) and B (green) reported for all 327 residues of each chain (**monA-B**), HHQ in chain B (red; **HHQ**) and focused on the putative catalysis-involved domains identified by comparative analysis with homologue KAS-III enzymes: adenosine binding site (**01_aBS**), palindromic "substrate-loop" (**02_sL**), helix H8-H9 loop (**03_h8-9**), hairpin-loop (**04_hL**), helix H12 (**h12**) and "oxyanion-loop" (**06_oL**).

Supplementary information REFERENCES

1. Kollman PA, Massova I, Reyes C, Kuhn B, Huo S, Chong L, Lee M, Lee T, Duan Y, Wang W, Donini O, Cieplak P, Srinivasan J, Case DA, Cheatham III TE: **Calculating structures and free energies of complex molecules: combining molecular mechanics and continuum models.** *Acc. Chem. Res.* 2000, **33**:889-897

2. Srinivasan J, Cheatham TE III, Cieplak P, Kollman PA, Case DA: **Continuum solvent studies of the stability of DNA, RNA, and phosphoramidate-DNA helices.** *J Am Chem Soc* 1998, **120**:9401–9409

3. Case DA, Darden TA, Cheatham TE, Simmerling CL, Wang J, et al. Amber 11, University of California, San Francisco 2006.

4. Schmidtke P, Bidon-Chanal A, Luque FJ, Barril X. **MDpocket: open-source cavity detection and characterization on molecular dynamics trajectories.** *Bioinformatics* 2011, **27**:3276-3285

5. Baker NA, Sept D, Joseph S, Holst MJ, McCammon JA: **Electrostatics of nanosystems: application to microtubules and the ribosome.** *Proc. Natl. Acad. Sci. USA* 2001, 98:10037-10041
